# Presence–absence polymorphisms of single-copy genes in the stony coral *Acropora digitifera*

**DOI:** 10.1186/s12864-020-6566-4

**Published:** 2020-02-13

**Authors:** Shiho Takahashi-Kariyazono, Kazuhiko Sakai, Yohey Terai

**Affiliations:** 1 Department of Evolutionary Studies of Biosystems, SOKENDAI (The Graduate University for Advanced Studies), Shonan Village, Hayama, Japan; 2 Sesoko Station, Tropical Biosphere Research Center, University of the Ryukyus, 3422 Sesoko, Motobu, Okinawa, 905-0227 Japan

**Keywords:** Structural variation, Expression difference, Genome diversity

## Abstract

**Background:**

Despite the importance of characterizing genetic variation among coral individuals for understanding phenotypic variation, the correlation between coral genomic diversity and phenotypic expression is still poorly understood.

**Results:**

In this study, we detected a high frequency of genes showing presence–absence polymorphisms (PAPs) for single-copy genes in *Acropora digitifera*. Among 10,455 single-copy genes, 516 (5%) exhibited PAPs, including 32 transposable element (TE)-related genes. Five hundred sixteen genes exhibited a homozygous absence in one (102) or more than one (414) individuals (*n* = 33), indicating that most of the absent alleles were not rare variants. Among genes showing PAPs (PAP genes), roughly half were expressed in adults and/or larvae, and the PAP status was associated with differential expression among individuals. Although 85% of PAP genes were uncharacterized or had ambiguous annotations, 70% of these genes were specifically distributed in cnidarian lineages in eumetazoa, suggesting that these genes have functional roles related to traits related to cnidarians or the family Acroporidae or the genus *Acropora*. Indeed, four of these genes encoded toxins that are usually components of venom in cnidarian-specific cnidocytes. At least 17% of *A. digitifera* PAP genes were also PAPs in *A. tenuis*, the basal lineage in the genus *Acropora*, indicating that PAPs were shared among species in *Acropora*.

**Conclusions:**

Expression differences caused by a high frequency of PAP genes may be a novel genomic feature in the genus *Acropora*; these findings will contribute to improve our understanding of correlation between genetic and phenotypic variation in corals.

## Background

Presence–absence polymorphisms (PAPs) are one type of structural variation, which describes genomic regions that are present in one genome, but absent in another genome within a species. When a PAP region contains a gene, the PAP directly affects gene function because some individuals lack the genomic region containing the gene.

Presence-absence differences of genomic regions among individuals within cultivated or domesticated strains generally refer to “presence-absence variation”, and has been reported based on genome-wide analyses in many cultivated plants [1, 2] and domesticated animals [3]. For example, at least 180 single-copy genes are presence–absence variants in two maize inbred lines [4]. Eleven genes are potential presence–absence variants in domesticated silkworm strains (*Bombyx mori*) [3]. In a model plant, 105 single-copy genes are PAPs among *Arabidopsis thaliana* strains [5]. Presence–absence difference of a gene is expected to have phenotypic effects. For example, presence–absence variation for 10 genes explains differences in anticancer alkaloid levels in three opium poppy (*Papaver somniferum*) strains [6].

In wild eukaryotic populations, genome-wide analyses of PAPs were performed in two anther-smut fungi and PAPs were observed in 2 and 0.6% of the total contents of autosomal genes in two species [7]. Except for this PAP analyses in fungi, PAPs for only a small number of genes have been reported in wild populations. In the fruit fly *Drosophila melanogaster*, PAPs were detected in three genes by PCR and Southern blot experiments, and one of these three PAPs was also detected in *D. simulans* [8]*.* In the oyster *Crassostrea gigas*, a PAP of one immune-related gene was reported and the expression of this gene was accordant with the PAP pattern [9]. Although PAPs are expected to play a role in shaping genetic diversity [4], genome-wide analyses of PAPs within wild populations have been limited.

Corals are declining in response to environmental changes, such as ocean acidification and increasing seawater temperatures [10]. In a stony coral species, genomic regions associated with thermal tolerance have been reported [11]. This indicates some variation in environmental response based on genetic variation within a coral species. Therefore, understanding genetic variation, including PAPs, within a coral species can help reveal phenotypic variation that is essential for adaptive resiliency to changing environments.

The advance of high-throughput sequencing has enabled the assembly of the whole genome of *Acropora digitifera* [12]. In this species, a correlation between PAPs of fluorescent protein gene sequences and a fluorescent phenotype have been reported [13]. However, a genome-wide analysis of PAPs in corals has not been performed. Here, we performed a genome-wide analysis of PAPs of single-copy genes in the stony coral *A. digitifera.*

We focused on single-copy genes that have evolved under functional constraint for three reasons. First, this strategy simplified the analysis. When multiple-copy genes are similar to each other, reads from these genes are mapped to all genes with high similarity. Therefore, it is difficult to detect the absence of a multi-copy gene. Second, this strategy avoids the influence of functional compensation. For multi-copy genes, if one gene copy is absent, another gene with high similarity may compensate for the loss. In this case, the effect of the absence of a gene may be smaller than that of a single-copy gene. Third, gene annotation generally includes mis-annotation of genes due to gene prediction. To avoid using mis-annotated genes, we used genes that have evolved under functional constraint.

We detected PAPs in approximately 5% of single-copy genes. More than half of the PAP genes detected were specific to cnidarian lineages or Acroporidae or *Acropora*. Among all PAP genes in the genome assembly, roughly half were expressed in adults and/or larvae, and these expressed genes were differentially expressed among individuals depending on their presence or absence status. We also analyzed *A. tenuis,* the basal lineage in the genus *Acropora* [14, 15]*,* and found that PAPs are a common genomic trait in *A. tenuis*, suggesting that PAPs may be a general feature among the genomes of species in the genus *Acropora*.

## Results

### PAPs in *A. digitifera*

**Fig. 1:**
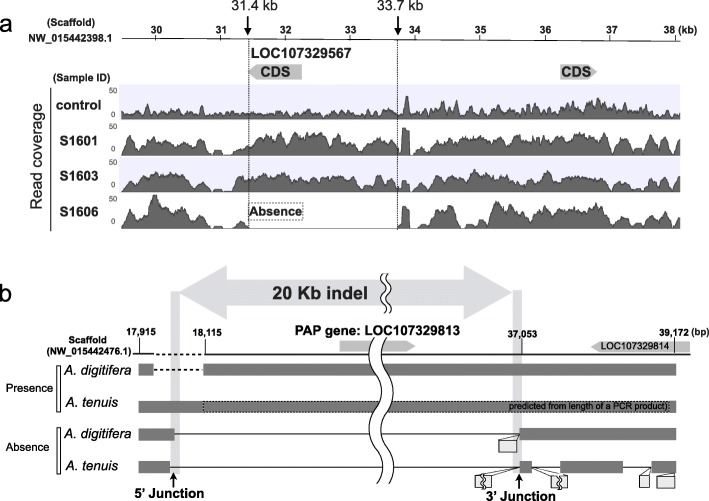
PAP in the *Acropora digitifera* genome. (a) An example of a PAP region lacking coverage. The genomic location of a CDS (LOC107329567) is shown with a gray arrow on the scaffold (NW_015442398.1). The read coverage (0 to 50) across the scaffold is shown in gray for a control and three samples. Dotted lines indicate the approximate start and end positions of the area with no read coverage. (b) Boundaries of absent alleles in *A. digitifera* and *A. tenuis*. The sequence from the scaffold (NW_015442476.1) was used as an *A. digitifera* present allele. The genomic locations of two genes are shown with light gray arrows on the line representing the scaffold sequence. Existing genomic regions are shown in dark gray in both present and absent alleles of *A. digitifera* and *A. tenuis*. The *A. digitifera* specific deletion is indicated by dashed lines. The sequence predicted from the length of a PCR product for the *A. tenuis* present allele is surrounded by a dashed line, insertions specific to absent alleles are shown by light gray boxes. The locations of the 5′ junction and 3′ junction are indicated by black arrows

We analyzed genome sequences of 11 *A. digitifera* (accession: DRR108003-DRR108012, DRR108024) that were collected in our previous study [13]. Genome sequences for each individual (4.2–11.6 Gb) were mapped to the *A. digitifera* genome assembly ver. 1.1. The average read coverage of CDSs based on only paired-end mapped reads for each individual ranged from 5.5 to 17.6 (see Additional file 1: Table S1). When we viewed the read coverage along the genome, we observed scattered regions of no or very low read coverage across the genomes of three individuals with mapping coverage over 9.5 (sample IDs: S1601, S1603, and S1606). For example, as shown in Fig. 1a, the read coverages were very low between positions 31.4 and 33.7 kb (scaffold NW_015442398.1) in the mapping results of an individual (sample ID: S1606), whereas control reads (used for *A. digitifera* genome assembly) were continuously mapped. The missing read coverage is explained by the absence of the genomic regions and thus a structural difference between individuals. We observed the absence of this region in 1 of 3 individuals (Fig. 1a), indicating a PAP. This genomic region included a gene with a CDS annotation (Fig. 1a), and this CDS was also identified as a PAP. Since the presence or absence of a gene may affect the function of the gene, we focused on PAPs of CDSs in the *A. digitifera* genome.

**Fig. 2:**
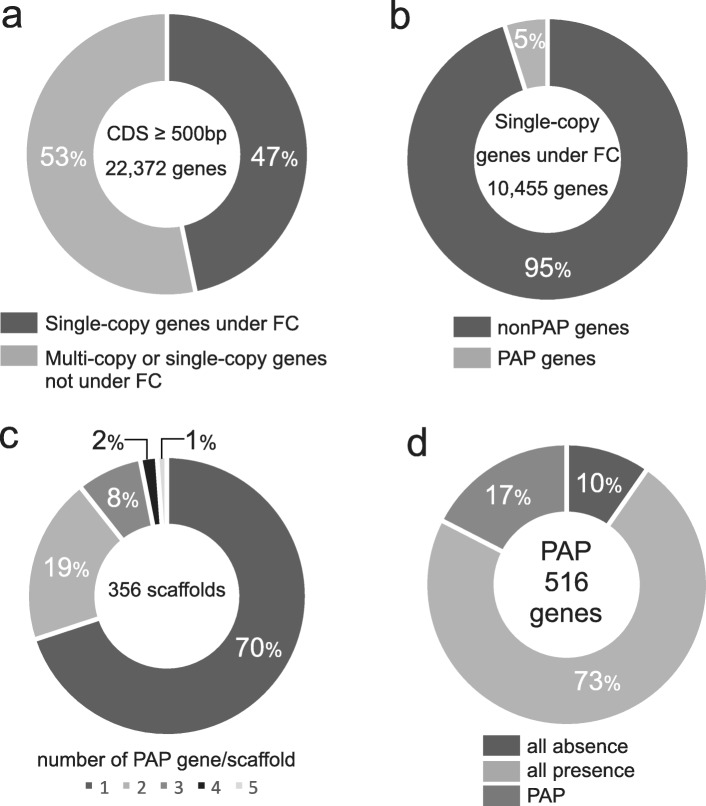
Characterization of PAPs in 33 *A. digitifera* and six *A. tenuis* individuals. (a) The frequencies of single-copy genes under functional constraint (47%: 10,455) and multiple-copy genes or single-copy genes without functional constraint (53%: 11,917) for all CDSs (22,372). (b) The frequencies of PAPs (5%: 516) and non-PAPs (95%: 9939) for single-copy genes under functional constraint. (c) The frequencies of PAPs on single scaffolds. (d) The frequencies of each presence–absence status for 516 genes (PAPs in *A. digitifera*) in *A. tenuis*

For all CDSs in the *A. digitifera* genome (22,372), we identified 10,455 single-copy genes under functional constraint (47%) (Fig. 2a). These single-copy genes were used for PAP identification. Our samples were collected from Sesoko, Okinawa (OI). OI belongs to the southern Ryukyu Archipelago composed by OI, Kerama (KIs), and Yaeyama, and sets of genome sequence reads of *A. digitifera* individuals from these regions were sequenced in Shinzato et al. 2015 [16]. Shinzato et al. [16] reported that *A. digitifera* individuals in the southern Ryukyu Archipelago show no population structure by model-based clustering analysis, although they were divided into four groups by principle component analysis. Using these data, we analyzed PAPs in 12 and 21 individuals with mapping coverage over 9.5 from OI and KIs respectively. We checked the population structure among these 33 individuals used for PAPs identification and eight individuals used for validation of PAPs by PCR (explain below) by fastSTRUCTURE [17]. As a result, the appropriate number of populations (K) to best explain the genetic differentiation was K = 1, suggesting no population structure in these individuals. We evaluated the absence of single-copy genes in each individual based on read coverage. An absence was defined as a CDS with no coverage of ≥80% of its total length. Among the 10,455 single-copy genes, 516 matched the criteria for PAPs used in this study, i.e., the absence of a gene in one or more individual. Surprisingly, PAPs accounted for approximately 5% of single-copy genes (Fig. 2b). We identified 32 transposable element (TE)-related genes in 516 PAPs. There was no significant correlation between number of PAP genes identified from an individual and mapping coverage (Additional file 1: Figure S1a), suggesting absence of a gene was not identified by low coverage of total number of reads. PAP genes are shown in Additional file 1: Table S2. Among 516 PAPs, 102 genes were identified as absent in only one individual and others were absent in two or more individuals (Additional file 1: Figure S1b). We selected three PAP genes that can be amplified by PCR, and verified the presence and absence of these genes by PCR (Additional file 1: Figure S2). The absent allele of a PAP region including two genes (LOC107336915–6) was sequenced, and an approximately 9 kb deletion in the absent allele was verified (Additional file 1: Figure S2g). Moreover, the PAP regions including one gene (LOC107329813) were amplified from *A. digitifera* and an outgroup species, *A. tenuis,* and the sequences of the boundaries of shared absent regions were determined (Fig. 1b, Additional file 1: Figure S2e and f). An absence consensus region spanning approximately 20 kb was identified from the sequence comparison between present and absent alleles from each of two species. A present allele from *A. digitifera* included an approximately 650-bp deletion at the 3′ boundary (Additional file 1: Figure S2e). In addition to verification of PAPs by PCR, we tried to determine sequences of absent alleles using long reads sequenced by a MinION sequencer using one individual (S1606). Although only 14,435 reads (> 1 kb) were determined (in total 31,402,994 bp), we found one read that covered an absent region (LOC107350576). The nucleotide sequence of this read is provided in Additional file 2. We aligned this read with the reference genome, and this alignment showed an approximately 4 kb deletion in the absent region (LOC107350576; Additional file 1: Figure S2h). According to these results, we verified that the genome of *A. digitifera* contains PAP regions.

To assess whether these PAPs are clustered in the genome, we verified their locations in the genome assembly. We found that 516 PAPs in the *A. digitifera* genome assembly were located on 356 scaffolds. In total, 70 and 19% of scaffolds included one and two PAPs, respectively (Fig. 2c). The maximum number of PAP genes on a single scaffold was five, and presence–absence patterns of these five PAP genes varied among 33 individuals (Additional file 1: Figure S3). Several combinations were shared in different subpopulations. These results suggest that PAPs are scattered throughout the genome.

Next, we analyzed the distribution of PAPs in two subpopulations (OI and KIs). Among 516 PAP genes, 357 were shared in two subpopulations (Additional file 1: Figure S4), and 49 and 110 were specific to OI and KIs, respectively (Additional file 1: Figure S4). In the KIs subpopulation, the number of subpopulation-specific PAPs was two-fold greater than that of the OI subpopulation. This high number was consistent with the large number of individuals used from KIs.

### Contribution of PAP genes to expression differences among *A. digitifera* individuals

**Fig. 3:**
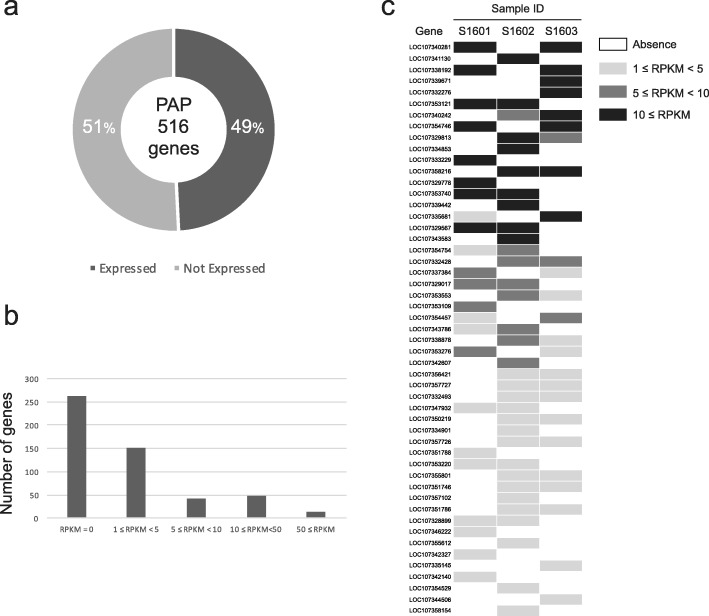
Expressions of genes showing PAPs. (a) The frequencies of expression (49%) and no expression (51%) for all PAP genes. (b) PAP genes were classified into five categories based on RPKM values: RPKM = 0, 1 ≤ RPKM < 5, 5 ≤ RPKM < 10, 10 ≤ RPKM< 50, 50 ≤ RPKM. The *y*-axis indicates the number of genes in each category. (c) Differences in gene expressions among individuals of *A. digitifera*. Rows are the 51 PAPs with complete matches between presence–absence patterns and expression patterns. Columns indicate the expression levels (RPKM) of three samples

To evaluate the effect of PAP genes on gene expression, RNA sequences from the same adult individuals of three *A. digitifera* (6.7 to 9.4 Gb; see Additional file 1: Table S1) and larvae (12.1 Gbp; accession: SRX1534820) were used to calculate normalized expression values (Reads Per Kilobase of exon model per Million mapped reads: RPKM) of 516 PAP genes. In 49% (254) of 516 PAPs, expression (RPKM ≥1) was detected in one or more *A. digitifera* adults and/or larvae (Fig. 3a), and 51% were not expressed at any individual or stage. Among 254 expressed PAP genes, 103 genes had RPKM values of greater than 5 (Fig. 3b).

Next, we identified PAP genes with complete correspondence between the presence–absence of genes and expression. The three individuals (sample ID: S1601, S1603, and S1606) for which RNA-seq data and genome sequence data (coverage ≥9.5) were available were used for this analysis. Both presence and absence individuals were observed among the three samples examined for 213 PAP genes. Among 213 genes, 83 genes were expressed in at least one individual. Among these 83 expressed genes, 62 genes were expressed in all present individuals (Additional file 1: Figure S5). The expression of 51 genes out of 62 corresponded with the presence–absence status of these genes (see Fig. 3c and Additional file 1: Table S3). In the remaining 11 genes among 62, an appearance of expression in absent individuals was observed. However, the expression of genes in absent individuals was caused by an artifact of RNA-seq reads being mapped to short parts in the absent regions. Sequences with high similarity to each of these short parts were found in the other regions in the genome, and RNA-seq reads originated from these similar sequences may be mapped to the short parts in the absent regions. The highest RPKM values in 18 genes exceeded 10, though individuals with a homozygous loss of alleles were not able to express these genes (Fig. 3c). Hence, PAP genes contribute to the expression differences observed among individuals of *A. digitifera*.

### What kind of genes become PAPs?

**Fig. 4:**
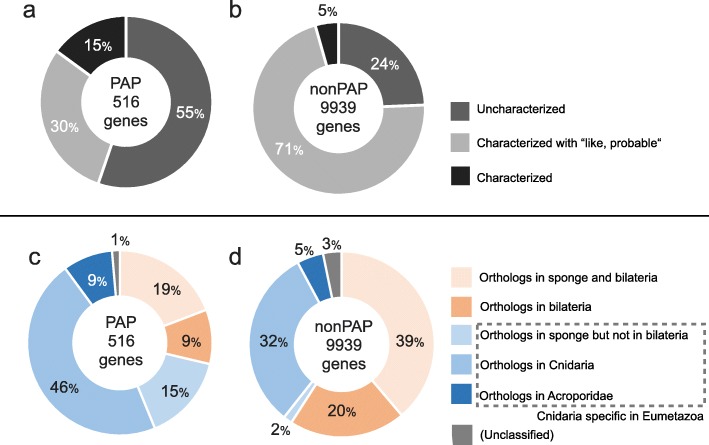
Characteristics of PAPs and non-PAPs. The frequencies of PAPs that were uncharacterized (55%), characterized with the suffix “-like or prefix “probable-” (30%), and annotated (15%) (a) and non-PAP genes (b). Distribution of the homologous genes for PAPs (c) and non-PAP genes (d)

To characterize PAPs, we first obtained descriptions of each polymorphic gene. Including TE-related genes, 55% (285 genes) were uncharacterized, 30% (154 genes) were annotated with the suffix “-like” or prefix “probable-”, and 15% (77 genes) were characterized with established gene names (Fig. 4a and Additional file 1: Table S2). By contrast, only 24% of non-PAP single-copy genes were uncharacterized (Fig. 4b).

We further collected information related to these genes from the literature. Only one gene has been reported in cnidarians. Potential toxic activity was suggested for one PAP gene (LOC107347179: endothelin-converting enzyme 1-like) based on a transcriptome analysis of the jellyfish tentacle [18, 19].

In addition, we analyzed the distribution of PAP genes by searching for orthologous genes in the following group of divergent animals: an ancestral species that belongs to the oldest diverged linage in metazoans, i.e., a sponge (*A. queenslandica*); 5 bilaterians, i.e., a fruit fly (*D. melanogaster*), vase tunicate (*C. intestinalis*), roundworm (*C. elegans*), Florida lancelet (*B. floridae*), and house mouse (*M. musculus*); 12 cnidarians except for Acroporidae, i.e., stony corals (*F. scutaria, P. strigosa, P. daedalea, M. cavernosa, S. hystrix, M. auretenra, S. siderea,* and *O. faveolata*), a starlet sea anemone (*N. vectensis*), sea anemones (*A. elegantissima* and *E. pallida*), and hydra (*H. vulgaris*); and 3 stony corals in Acroporidae (*M. aequituberculata, M. digitata, and A. tenuis*). For comparison, we also searched for non-PAP single-copy genes in the same animals. For searches against non-cnidarian animals, we identified orthologous genes for 59% of non-PAP genes: 39% in both a sponge and bilaterians and 29% in at least one or more bilaterians. However, we identified orthologous genes for only 28% of PAP genes (19% in both a sponge and bilaterians and 9% in bilaterians, in total 148 genes) (Fig. 4c and d). Among these 148 genes, 127 were uncharacterized or hypothetical proteins. In the search against cnidarians, we found orthologous genes for 32% of non-PAP genes, and 5% existed only in Acroporidae or *Acropora* (Fig. 4d). For 46% of PAP genes, orthologous genes were found in cnidarians except Acroporidae, and 9% existed only in Acroporidae or *Acropora* (Fig. 4c). We detected 70% PAP vs. 39% non-PAP orthology for genes only found in cnidarian linages that included genes lost in bilaterian linages but present in a sponge. (Fig. 4c and d, bluish colors).

### Absent genes in the *A. digitifera* genome assembly

PAPs in the *A. digitifera* genome raised the possibility that the genome of the individual used for genome assembly contains the absent allele of PAPs. In other words, there may be missing genes in the reference genome sequences. To identify missing PAP genes, reads that were not mapped to the *A. digitifera* genome sequence were collected for three samples (S1601, S1603, and S1606). After removal of reads that originated from symbiotic algae, the remaining reads were assembled into contigs and open reading frames were predicted. Using this approach, we identified 43 new single-copy genes under functional constraint (Additional file 1: Table S4). Among these 43 genes, 2 were present in all 33 individuals and 41 were PAPs in 33 individuals. These results suggest the possibility that one single reference genome of *A. digitifera* may underestimate the total number of genes in the genome of this species.

### Shared presence–absence polymorphisms in *A. digitifera* and *A. tenuis*

To evaluate whether PAPs were common in *Acropora* species, we analyzed *A. tenuis,* the basal lineage in the genus *Acropora*. Genome sequences of six *A. tenuis* larvae (8.1–10.5 Gb) were determined using the Illumina HiSeq2500 platform (Additional file 1: Table S1). The percentage of genomic intervals with no coverage for each of 516 *A. digitifera* PAPs was calculated. Although we used only six individuals, we detected 17% (90) of 516 *A. digitifera* PAPs in *A. tenuis*. We found that 73% (376) and 10% (50) of 516 *A. digitifera* PAPs were present and absent in all six individuals, respectively (Fig. 2d).

Next, we analyzed whether these shared PAPs were present in the common ancestor of *Acropora* species or if such events occurred independently. We determined the sequences at the boundary positions of an absence region in one PAP (LOC107329813) from *A. digitifera* and *A. tenuis*. The boundary positions for the two species were nearly identical (Fig. 1b; Additional file 1: Figure S2e and f), indicating a common ancestral origin of this PAP. In this analysis, we used only one PAP gene region. Therefore, to reveal the proportion of PAP genes that originated from the common ancestor of *Acropora* species, further analyses using large numbers of PAP loci are required.

## Discussion

### PAPs account for 5% of single-copy genes and contribute to expression differences among *A. digitifera* individuals

Among various types of genetic variation, presence–absence variation of genes may have a particularly large effect on phenotypes because the absence of a gene is equivalent to a “loss of function” of the gene. When mutations, insertions, and deletions in a gene cause a loss of function, these genetic changes are typically deleterious. In particular, the absence of a single-copy gene may have a greater effect than the absence of a multiple-copy gene because paralogs have the potential for functional compensation. In wild populations, deleterious alleles (in this case, an absent allele), are expected to be immediately or rapidly removed by natural selection (negative selection). However, in cultivated plants, domesticated animals, and model organisms, these alleles can be maintained and, as a consequence, the functional constraint on the gene may be relaxed. Indeed, presence–absence difference of single-copy genes have been found in cultivated plants [4] and model plant strains [5].

We considered the potential deleterious effects of PAPs observed in this study. However, we found evidence suggesting that the absence of alleles is not highly deleterious. First, our samples and sequence data from the published database [16] were obtained from adult individuals; these individuals developed without serious defects, suggesting that the homozygous absence of these alleles was not lethal. Second, the frequencies of the homozygous absence allele were relatively high in PAPs. Among 516 PAP genes, 414 exhibited a homozygous absence in two or more individuals out of 33 total individuals. Third, the polymorphic state of over half of the PAP genes was shared among two subpopulations. If the homozygous absence was deleterious, individuals lacking both copies should be removed from the population by purifying selection, minimizing shared PAPs among populations. Accordingly, we concluded that most PAPs were not deleterious or were only slightly deleterious.

Next, we considered the possibility that the PAP genes did not have function in corals and therefore were evolutionarily neutral. All genes analyzed in this study were single-copy genes under functional constraint. In particular, we detected that 43% of PAP genes in a sponge or bilaterians were orthologous, suggesting that these genes were conserved during the evolution of metazoans or eumetazoa. Hence, a substantial number of PAP genes were likely functional.

A notable feature of the PAPs was that the present alleles were expressed. Among 516 PAPs in the *A. digitifera* genome assembly, 254 genes were expressed, and the RPKM values of 18 genes exceeded 10. However, despite such high expression of present alleles, individuals with a homozygous absence did not exhibit expression. Among 83 expressed PAP genes in three individuals, the presence–absence patterns were consistent with expression patterns for 51 genes. The patterns for PAPs were not linked with each other, and thus there was variation in the combinations of expressed genes among individuals (Fig. 3c). In 21 PAP genes out of 83, we detected both an individual with expression of a present allele and an individual with no expression of a present allele. This variation may be explained by the regulation of gene expression. Hence, PAPs contribute to gene expression differences among *A. digitifera* individuals.

The PAP genes without expression in adult and larval stages (262 genes) have evolved under functional constraint, suggesting that these genes are expected to be functional. One possibility to explain PAP genes without expression is that these genes may express in a short time period during a life cycle in *A. digitifera*, such as certain developmental stages, a reproduction stage, and a seasonal response. The other possibility is a stress response. These genes may express response to various stress such as high temperature, acidification, irradiation of UV light, and a physical damage.

### Limited distribution of PAP genes in cnidarian lineages

In general, single-copy genes are assumed as essential for viability and the persistence of species. However, PAPs accounted for 5% of single-copy genes under functional constraint in *A. digitifera*. This observation prompted various questions, e.g., what are the functions of genes showing PAPs and how are these genes maintained during the evolution of cnidarians? To address these questions, we examined the putative gene functions for PAP genes. Among PAP genes, 55% were uncharacterized, whereas only 24% of non-PAP single-copy genes were uncharacterized. Gene functions were uncharacterized when a similar gene with a known or predicted function did not exist in the public database; genes with information deposited in databases may be biased toward model organisms and limited in cnidarians. In other words, the uncharacterized genes may be *Acropora* or Acroporidae or cnidarian specific. Instead of searching for gene functions, we examined the distribution of PAP genes in cnidarian and non-cnidarian lineages. Among all PAPs, 70% of the genes were distributed to only cnidarian lineages (*Acropora* or Acroporidae or other cnidarian lineages) or lost in bilaterian linages (present in a sponge). The limited distribution of PAP genes in cnidarian lineages suggests that these genes may be related to traits specific to cnidarians.

A well-known trait specific to cnidarians is the cnidocyte, which usually contains venom [20]. Indeed, we found four candidate toxin genes in PAP regions (highlighted in gray, Additional file 1: Table S2) and one gene associated with toxic activity (LOC107347179: endothelin-converting enzyme 1-like) that has been reported in jellyfish [18, 19]. Among these four candidate genes, three were expressed in adults. The sequence diversity of toxins is important to minimize the development of tolerance against toxins [21], and presence–absence variation of venom proteins within species has been reported by a proteomics-based approach in snakes [22, 23]. The PAP of toxin genes may generate toxin diversity among individuals in *A. digitifera*. Although a multi-gene family of toxins has been reported in *A. digitifera* [24], these genes were not analyzed in this study.

### PAPs are characteristic features in genomes of the genus *Acropora*

*A. tenuis* is located at the basal position in the genus *Acropora* [14, 15]. At least 17% of *A. digitifera* PAP genes were also PAPs in *A. tenuis*, suggesting that these PAPs are a general genomic characteristic in the genus *Acropora*, though the PAPs were only analyzed in two species belonging to the genus. For one gene, the boundaries of presence and absence alleles were nearly identical in these two species, indicating a single origin of the absence allele and the persistence of the PAP during the evolution of the genus *Acropora*. This analysis of boundary positions was limited to a single gene owing to gaps in the reference genome, the long length of deleted regions, and genetic divergence between species. Therefore, we could not rule out the possibility of independent acquisitions of other PAPs in *A. tenuis*.

## Conclusions

Coral reefs have decreased due to environmental changes, such as increasing water temperatures and ocean acidification [10]. Genetic variation that affects the phenotypes of individuals may play an important role in adaptation to changing environments [11]. In this study, we detected PAPs in single-copy genes under functional constraint in *A. digitifera*. The features of these PAP genes, such as the expression of present alleles, distribution in cnidarians, and conservation of sequences among *Acropora* or Acroporidae species, indicate potential functional roles related to the *Acropora* or Acroporidae species or cnidarians. Although we do not have direct evidence that PAPs are responsible for phenotypic differences, they generate expression differences among individuals that may be associated with phenotypic differences among them. The functional analysis of PAP genes found in this study may provide insight into the role of these genes for phenotypic differences of corals.

## Methods

### Specimen collection and species identification

The collection of 11 *A. digitifera* is explained in our previous study [13]. In brief, a branch fragment was collected from each of 11 *A. digitifera* colonies in Sesoko, Okinawa, Japan. Gametes from four *A. tenuis* were collected in the field in front of Sesoko Station (Tropical Biosphere Research Center, University of the Ryukyus). Gamete-collecting devices were set above each individual colony, and bundles of gametes were brought to the laboratory and mixed to allow fertilization. Larvae were reared by daily transfer to fresh seawater and maintained at approximately 26 °C. Six larvae of *A. tenuis* were preserved in RNAlater (Waltham). Species were identified based on morphology by Dr. Kazuhiko Sakai for *A. digitifera* and by Dr. Masayuki Hatta for *A. tenuis*. Collections of samples were approved by the Aquaculture Agency of Okinawa Prefecture (permit numbers 28–31 and 27–1).

### DNA and RNA extraction and sequencing

Methods used for 11 *A. digitifera* is explained in our previous study [13]. Genomic DNAs were extracted from six larvae of *A. tenuis* using DNeasy Blood & Tissue Kits (QIAGEN, Hilden, Germany) and were used for polymerase chain reaction (PCR) and the construction of DNA libraries. Following the manufacturer’s instructions, DNA libraries of six *A. tenuis* were constructed using the NEBNext Ultra II DNA Library Prep Kit (Illumina, Inc., San Diego, CA, USA).

In high-throughput DNA sequencing, short DNA sequences (paired-end, 125 bp) were determined from the libraries using the Illumina HiSeq2500 platform (Illumina). The accession numbers of DNA reads are shown in Additional file 1: Table S1.

### PAP identification and detection

To identify PAPs in *A. digitifera*, coding sequences (CDSs) of single-copy genes were evaluated. Since the genes annotated by gene prediction include a possibility of mis-prediction, we used genes under functional constraint. The schematic representation of identification of single-copy genes under functional constraint are shown in Additional file 1: Figure S6. For the first step, sequences of less than 500 bp and isoform sequences other than the longest isoform were removed from CDSs of the publicly available *A. digitifera* genome assembly ver. 1.1. The remaining CDSs were used to generate a database, and the same CDSs were used as queries for blastn searches [25]. A CDS with high similarity (cut-off *e*-value = 1e^− 10^) to only itself was considered a single-copy gene. CDSs with regions of no read coverage (explained below) exceeding 80% of the region in reads (DRR001426_1 and DRR001427_1) used for the assembly of the *A. digitifera* genome were removed from single-copy genes. In the second step, single-copy genes were used as queries for tblastn searches [25] and a database (Cnidaria database 1) was generated using CDSs of one species of Hydrozoa, *Hydra vulgaris* [26]; three species of Actiniaria, *Nematostella vectensis* [27], *Anthopleura elegantissima* [28], and *Exaiptasia pallida* [29]; and eight Scleractinia except for Acroporidae, *Madracis auretenra*, *Orbicella faveolata*, *Pseudodiploria strigosa*, *Platygyra daedalea*, *Seriatopora hystrix*, *Montastraea cavernosa, Fungia sctaria* [28], and *Siderastrea siderea* [30]. RefSeq Assembly IDs or URLs of all species are shown in Additional file 1: Table S5. A single-copy gene of *A. digitifera* with high similarity (cut-off *e*-value = 1e^− 50^, identity ≥50%) to a CDS in Cnidaria database 1 was considered a single-copy gene under functional constraint. In the third step, a single-copy gene of *A. digitifera* without high similarity in the previous step was used as a query for downstream tblastn searches [25]. CDSs of three Acroporidae species, *Montipora aequituberculata, M. digitata* [31], and *Acropora tenuis* were used to generate a database (Cnidaria database 2). A single-copy gene of *A. digitifera* with high similarity (cut-off *e*-value = 1e^− 50^, identity ≥50%) to a CDS in Cnidaria database 2 and a highest similarity sequence from database 2 were retained for further analysis. The rates of synonymous (dS) and nonsynonymous (dN) substitutions along an alignment of an *A. digitifera* CDS and its highest similarity were estimated using the Nei-Gojobori method [32]. dN and dS values were estimated using MEGA-CC software [33]. *A. digitifera* CDSs under purifying selection (dN < dS, *P* < 0.05) were considered a single-copy gene under functional constraint. Finally, CDSs that were regarded as single-copy genes under functional constraint in the second and third steps were used for PAP identification.

Sequence reads (paired-end, 125 bp) from the genomic DNA libraries of 11 *A. digitifera* individuals were mapped to the *A. digitifera* genome assembly ver. 1.1 using CLC Genomics Workbench (https://www.qiagenbioinformatics.com/). Reads showing high similarity (> 90%, > 112 bp) were mapped to query sequences. The reads mapped in pairs were used to calculate the read coverage at each site using CLC Genomics Workbench. Three individuals with mapping coverage over 9.5 (IDs: S1601, S1603, and S1606) were used for PAP identification and the remaining eight individuals were used only for validation of PAPs by PCR (explained below). The proportion of each CDS with no coverage was calculated. As a control, the same analysis was performed using reads (accession: DRR001426_1 and DRR001427_1) used for the assembly of the *A. digitifera* genome. In addition to 3 *A. digitifera* individuals (IDs: S1601, S1603, and S1606), the publicly available genomic DNA reads of *A. digitifera* collected from the southern Ryukyu Archipelago located in southwestern Japan were used. Individuals with read mapping coverage over 9.5 were selected. Genomic DNA reads of 9 and 21individuals collected from Okinawa Island (OI) and Kerama Islands (KIs) respectively were downloaded from DNA Data Bank of Japan (DDBJ). The accession numbers for genomic DNA reads are shown in Additional file 1: Table S6. In total, 33 sets of genomic DNA reads of 12 and 21 individuals collected from OI and KIs were analyzed in this study. CDSs with regions of no read coverage exceeding 80% of the region were considered absent from the genome. When a gene was lacking in the genome of one or more individuals, it was identified as a PAP gene. Twelve genes were annotated in *A. digitifera* genomic DNA (ver. 1.1), but the reads from all 33 individuals were not mapped (no read coverage < 20%) to the CDS regions. We removed these 12 genes from our analyses. Annotations of PAP and non-PAP genes were obtained by blastn searches [34] (*e*-value = 1e^− 6^) to non-redundant sequences in GenBank (NCBI). Genes with transposable element (TE)-related descriptions were considered TE-related genes. One notable feature of our PAP detection method is that an absence is strictly defined as a homozygous lack of a gene.

### Estimation of the number of populations among 41 *A. digitifera* individuals

SNPs (867,817) located on synonymous sites were extracted using CLC Genomics Workbench (https://www.qiagenbioinformatics.com/) from mapping results (explained in PAP identification and detection) of 33 individuals used for PAPs identification and eight individuals used for validation of PAPs by PCR. The cutoff of the minor allele frequency was set as 2% to remove singletons from the dataset. The appropriate number of populations (K) were estimated by fastSTRUCTURE [17]. The K values ranged from 1 to 5. The Python script chooseK.py, provided with fastSTRUCTURE [17], was used to identify the K value.

### Validation of PAPs by PCR and cloning of DNA sequences in absent regions

Two loci containing three PAPs (LOC107329813, LOC107336915, and LOC107336916) were selected for validation by PCR. Two genes (LOC107336915–6) were located in tandem and one primer set was used for both genes. For two loci, the existence of a PAP was verified by PCR using primer sets (29813_LF1/29813_LR1 and 36916_LF1/36916_LR1) located up- and downstream of the absent region. Using these primer sets, short PCR products (expected size, approximately 5 kb) were expected to be amplified when alleles were absent, and no PCR products (because the fragment was too long to amplify; expected size, approximately 14–22 kb) were expected when alleles were present. To confirm the annealing of primers for the validation of PAPs, one or two primers (29813_checkF1, 29813_checkR1, and 36916_checkF2) were designed in the gene region (presence) for each PAP. The primer sequences are shown in Additional file 1: Table S7.

To check quality of genomic DNA from each individual, a primer set (34639_F/34639_R) was designed to amplify ~ 3-kb PCR products (elongation factor 1-alpha: LOC107334639) as a positive control. Primer positions and sequences are given in Additional file 1: Figure S2 and Additional file 1: Table S7. PCR was performed using GeneAmp PCR System 9700 (Applied Biosystems, Carlsbad, CA, USA), and Ex Taq DNA Polymerase (TaKaRa, Shiga, Japan) was used for all PCRs. Genomic DNAs extracted from 11 *A. digitifera* individuals (sample ID: S1601–8 and S1610–12) were used as templates for PCRs. Reaction conditions for the amplification of the absent region in PAPs and the positive control were as follows: denaturation for 3 min at 93 °C, followed by 30 cycles of denaturation for 1 min at 93 °C, annealing for 1 min at 63 °C, and extension for 5 min at 72 °C. To confirm the annealing of primers for the validation of PAPs (primer sets; 29813_checkF1/29813_LR1 and 36916_checkF2/36916_LR1), the same conditions described above were used. For other primers to confirm the annealing of primers for the validation of PAPs (primer sets; 29813_LF1/29813_checkR1), the PCR conditions were as follows: denaturation for 3 min at 93 °C, followed by 30 cycles of denaturation for 1 min at 93 °C, annealing for 1 min at 60 °C, and extension for 2 min at 72 °C.

To determine the boundaries of large insertions and deletions (indels) including a gene between presence and absence alleles, sequence determination was performed from three PAPs (LOC107329813, LOC107336915, and LOC107336916). For LOC107336915–6, the up- and downstream sequences of an indel in the absent allele was amplified by PCR using the primer set 36916_LF1/36916_LR1. The genomic DNA of *A. digitifera* (sample ID: S1601) was used as a template. PCR conditions were as follows: denaturation for 3 min at 93 °C, followed by 40 cycles of denaturation for 1 min at 93 °C, annealing for 1 min at 63 °C, and extension for 5 min at 72 °C. The sequence of the PCR product was determined using the Applied Biosystems Automated 3130xl Sequencer with the following primers: 36916_LF1, 36916_sF1, 36916_sF2, 36916_sF3, 36916_sF4, 36916_sF5, 36916_LR1, 36916_sR1, 36916_sR2, and 36916_sR3. The determined sequence was aligned with the reference sequence (*A. digitifera* scaffold: NW_015441149.1) using MEGA ver. 7 [35], and manually.

For LOC107329813, the up- and downstream sequences of an indel in the absent allele was amplified by PCR using the primer set 29813_LF1/29813_LR1. The genomic DNAs of *A. digitifera* (sample ID: S1604) and *A. tenuis* (sample ID: T3) were used as templates. PCR conditions were as follows: denaturation for 3 min at 93 °C, followed by 30 cycles of denaturation for 1 min at 93 °C, annealing for 1 min at 63 °C, and extension for 5 min at 72 °C. PCR products were cloned into the T-Vector pMD20 vector (Takara), and the sequences were determined using the Applied Biosystems Automated 3130xl Sequencer with the following primers: 29813_F2, 29813_F6dig, 29813_R2, 29813_R5, and 29813_R6dig for *A. digitifera,* and 29813_F2, 29813_F5, and 29813_R3-R4, 29813_R6-9ten for *A. tenuis*. The upstream sequence of an indel in the present allele of *A. tenuis* (sample ID: T8) was amplified using the primer set 29813_F2/29813_checkR3, and PCR conditions were as follows: denaturation for 3 min at 93 °C, followed by 30 cycles of denaturation for 1 min at 93 °C, annealing for 1 min at 55 °C, and extension for 1 min at 72 °C. The downstream sequence of an indel in the present allele of *A. tenuis* (sample ID: T8) was amplified using the primer set 29813_checkF1/29813_LR1, and PCR conditions were as follows: denaturation for 3 min at 93 °C, followed by 30 cycles of denaturation for 1 min at 93 °C, annealing for 1 min at 60 °C, and extension for 2 min at 72 °C. These sequences were verified using the Applied Biosystems Automated 3130xl Sequencer. All determined sequences were aligned with the reference sequence (*A. digitifera* scaffold: NW_015442476.1) using MEGA ver. 7 [35], and manually.

### Determination of DNA sequences in absent regions by nanopore sequencing

Genomic DNA was extracted from a coral fragment of *A. digitifera* (sample ID: S1606) using MagAttract HMW DNA Kit (QIAGEN) and was used for the construction of a MinION sequencing library. Following the manufacturer’s instructions, a sequencing library was constructed using the 1D2 Sequencing Kit (Oxford Nanopore Technologies, Oxford, UK). The sequencing library was loaded onto the R9.5 flowcell (Oxford Nanopore Technologies) using a Library Loading Bead Kit (Oxford Nanopore Technologies). Sequencing was performed with a MinION sequencer (Oxford Nanopore Technologies). After sequencing, read data were base-called using Albacore v2.3.3 (Oxford Nanopore Technologies) and adapter sequences were trimmed from reads using Porechop v0.2.3 (https://github.com/rrwick/Porechop). Reads were filtered by the minimum average read quality score (5) and minimum read length (1000 bp) using NanoFilt v2.2.0 (https://github.com/wdecoster/nanofilt). Reads that passed this filter were used for the following analysis.

MinION reads that could cover both up- and downstream sequences of an indel in an absent allele were searched by blastn with default settings. MinION reads were used as a database and up- and downstream sequences (around 200 bp) of indels in absent alleles observed in a coral individual (sample ID: S1606) were used as query. When both up- and downstream queries hit to the same read, this read was aligned with the *A. digitifera* scaffold that included a targeted absent allele using MEGA ver. 7 [35], and manually.

### Contribution of PAP genes to expression differences among individuals

PAP genes that were expressed were identified using RNA-seq reads from previous studies. RNA-seq reads (paired-end, 125 bp) from each of three adult individuals (sample IDs: S1601, S1603, and S1606) of *A. digitifera* and larvae (12.1 Gbp; accession: SRX1534820) (Table S1) were mapped to the *A. digitifera* genome assembly ver. 1.1. Reads showing similarity (90%) with 90% read lengths were mapped to reference sequences and expression levels were calculated using CLC Genomics Workbench. RPKM (Reads Per Kilobase of exon model per Million mapped reads) were used as normalized expression values. PAP genes with RPKM ≥1 in at least one adult and/or larva were considered expressed genes.

### Identification of single-copy PAP genes that are absent in the *A. digitifera* genome assembly

Reads of three *A. digitifera* (sample IDs: S1601, S1603, and S1606) that were not mapped to the reference genome were used for this analysis (details are described in the PAP Identification and Detection section). To remove the reads that originated from symbiotic algae in corals, reads showing a similarity of > 80% with a length of > 100 bp were mapped to the genomes of three species in the family Symbiodiniaceae [36]; *Fugacium kawagutii* [37], *Breviolum minutum* [38], and *Symbiodinium microadriaticum* [39], and one transcriptome of *Cladocopium goreaui* [40] using CLC Genomics Workbench and unmapped reads were collected. The reads from each of three individuals that were not mapped to coral and algal genomes or a transcriptome were assembled into contigs, independently.

Assembled contigs were first filtered by open reading frame prediction. RNA-seq reads from each of three *A. digitifera* (sample IDs: S1601, S1603, and S1606) were mapped to assembled contigs from each of three individuals, respectively, using TopHat 2.1.0 [41]. RNA-seq reads from *A. digitifera* larvae (accession: DRR054773) were also mapped to assembled contigs. Based on adult and larval RNA-seq mapping, transcriptomes of newly-assembled contigs from each of three individuals were predicted using Cufflinks 2.1.1 [42]. CDSs in assembled contigs were annotated using TransDecoder 3.0.1 (https://github.com/TransDecoder/TransDecoder/wiki) and complete CDSs longer than 500 bp were used for the following analysis. As a second filter, the same analysis used for identification of genes under functional constraint was performed with CDSs in assembled contigs, and CDSs under functional constraint were retained for the third filter. As a third filter to select single-copy genes, these assembled CDSs from an individual were used as query for blastn searches [25]. *A. digitifera* CDSs (excluding sequences shorter than 500 bp and isoform sequences other than the longest isoform) and newly-assembled CDSs from an individual were used to generate a blast database. A CDS with high similarity (cut-off *e*-value = 1e^− 10^) to its own CDS was considered a single-copy gene. This process was performed with each of the three assembled CDSs from three individuals. To remove overlaps of genes between the three assembled CDSs, reciprocal blastn searches [25] were performed between single-copy genes in the three newly-assembled CDSs. When a gene had a reciprocal hit, only one sequence was retained. As the fourth filter, control reads (accession: DRR001426_1 and DRR001427_1) were mapped to the assembled single-copy genes using CLC Genomics Workbench. Reads showing similarity (90%) with 90% read lengths were mapped to query sequences. Genes with regions of no read coverage exceeding 80% of the region were considered absent from the genome of an individual used for *A. digitifera* genome assembly and retained as newly-identified single-copy genes.

Newly-identified single-copy genes were annotated by blastn searches [34] (*e*-value = 1e^− 6^) against non-redundant sequences in GenBank (NCBI). These newly-identified single-copy genes were used for PAP identification. To identify PAPs in new single-copy genes, reads for each of the 33 *A. digitifera* individuals, described above were mapped to the newly-identified single-copy genes using CLC Genomics Workbench. Reads showing similarity (90%) with 90% read lengths were mapped to query sequences and the same procedure used for the identification of PAPs was performed.

### Identification of orthologs of *A. digitifera* single-copy genes in other animal species

A Bilateria database for blast searches was constructed from predicted protein sequences of five species: *Drosophila melanogaster* [43], *Ciona intestinalis* [44], *Branchiostoma floridae* [45]*, Caenorhabditis elegans* [46], and *Mus musculus* [47]. The single-copy genes in *A. digitifera* were translated into protein sequence and used as queries in blastp searches [25]. The top hit with *e*-value ≥1e^− 30^ and identity ≥30% was regarded as an orthologous gene. Orthologous genes of *A. digitifera* single-copy genes in *Amphimedon queenslandica* (sponge) [48] were searched by blastp [25]. The blastp search [25] parameters and procedure for the identification of orthologous genes were the same as those used in the former search. Orthologous genes of *A. digitifera* single-copy genes in Cnidaria and Acroporidae were searched by tblastn [25] as explained in the section on PAP identification and detection.

### Validation of PAPs in *A. tenuis*

After the removal of the adaptor sequences and low-quality reads, genomic DNA sequence reads (paired-end, 125 bp) from the DNA libraries of six *A. tenuis* individuals were mapped to the *A. digitifera* genome assembly ver. 1.1 using CLC Genomics Workbench. Reads showing high similarity (> 90%, > 100 bp) were mapped to reference genome sequences. The same analysis used for PAP identification was repeated for six *A. tenuis* individuals.

## Supplementary information

**Additional file 1 Figure S1.** Number of PAP genes in 33 *A. digitifera* individuals. (a) The number of PAP genes (y axis) were plotted against mapping read coverage (x axis) for 33 individuals. (b) The number of PAP genes that were identified as absent homozygous in 1 to 33 individuals. The x-axis represents the numbers of absent homozygous individuals comparing with *A. digitifera* genome (ver. 1.1) for each PAP gene. The y-axis represents the number of PAP genes. 20% (102) PAP genes appeared in only one individual, and the remaining 80% (*n* = 414) appeared more than once. **Figure S2.** Electrophoresis patterns of PAPs among 11 *A. digitifera* individuals and the structures of PAP loci. The results of PCR products of (a) LOC107329813 and (b) LOC10736915–6. Positions of primers are indicated by arrows, under the schematic representations of genes. Primer names are given under each arrow. M1 and M2 indicate the molecular markers, φX174 HaeIII digest and λ-HindIII digest, respectively. Sample IDs S1601-S1612 are indicated as 01–12. NC indicates negative control. The state of presence (P) and absence (A) estimated by the no coverage region are also shown above the photos. Presence state included both presence homozygous and presence and absence heterozygous samples. In the central panel of (a), one sample (S1603) was expected as presence with a PCR product of present region. However, no PCR product was amplified. (c) The result of PCR for positive control to check the quality of genomic DNA of each sample. (d) Positions of primers for sequence determination of the absent region in LOC107329813. Alignments of 5′ junction sequences and 3′ junction sequences from present and absent alleles are shown in (e) and (f), respectively. (g) Positions of primers for sequence determination of the absent region in LOC10736915–6. (h) Boundaries of absent alleles shown by MinION reads. **Figure S3.** Combination of presence and absence status of five PAP genes located on one scaffold. Schematic representations of a scaffold (NW_015441940.1) with gene positions (arrows) shown at the top. Rows indicate 33 individuals from two subpopulations. Columns are PAP genes, and the positions are shown by gray arrows at the top. Presence and absence status is shown by white and grey, respectively. **Figure S4.** The Venn diagram of PAP genes identified in two subpopulations. The numbers of PAP genes specific to subpopulations and shared among subpopulations are in the Venn diagram. **Figure S5.** Expressions of genes showing PAPs in three individuals. **Figure S6.** The schematic representation of identification of single-copy genes under functional constraint. **Table S1.** Overview of high-throughput sequencing. **Table S2.** Gene IDs and descriptions of PAP genes identified in 33 *A. digitifera*. **Table S3.** Percentage of no coverage region and RPKM of 51 PAP genes. **Table S4.** Description and no coverage (%) of newly-identified single-copy genes. **Table S5.** Genome or transcriptome data used for blast databases in this study. **Table S6.** Information for high-throughput sequencing data. **Table S7.** Primer sequences.

**Additional file 2.** A nucleotide sequence for validation of PAPs.

## Data Availability

The data sequenced in this study were deposited in the DNA Data Bank of Japan (DDBJ) Sequenced Read Archive under accession numbers DRR110778-DRR110783. A nucleotide sequence for validation of PAPs is provided in the Additional file 2. The accession numbers and web links to the respective data analyzed in this study are listed in Tables S1, S5, and S6.

## Abbreviations

CDSs: Coding sequences
DDBJ: DNA Data Bank of Japan
KIs: Kerama Islands
OI: Okinawa Island
PAPs: Presence–absence polymorphisms
PCR: Polymerase chain reaction
RPKM: Reads per kilobase of exon model per million mapped reads
TE: Transposable element
