# Supplementary material for: Presence–absence polymorphisms of single-copy genes in the stony coral Acropora digitifera
Source: BMC Genomics. 2020 Feb 13;21:158. doi: 10.1186/s12864-020-6566-4 (PMC7020367; doi:10.1186/s12864-020-6566-4)
Supplement: Supplementary file 1 — Additional file 1 Figure S1. Number of PAP genes in 33 A. digitifera individuals. (a) The number of PAP genes (y axis) were plotted against mapping read coverage (x axis) for 33 individuals. (b) The number of PAP genes that were identified as absent homozygous in 1 to 33 individuals. The x-axis represents the numbers of absent homozygous individuals comparing with A. digitifera genome (ver. 1.1) for each PAP gene. The y-axis represents the number of PAP genes. 20% (102) PAP genes appeared in only one individual, and the remaining 80% (n = 414) appeared more than once. Figure S2. Electrophoresis patterns of PAPs among 11 A. digitifera individuals and the structures of PAP loci. The results of PCR products of (a) LOC107329813 and (b) LOC10736915–6. Positions of primers are indicated by arrows, under the schematic representations of genes. Primer names are given under each arrow. M1 and M2 indicate the molecular markers, φX174 HaeIII digest and λ-HindIII digest, respectively. Sample IDs S1601-S1612 are indicated as 01–12. NC indicates negative control. The state of presence (P) and absence (A) estimated by the no coverage region are also shown above the photos. Presence state included both presence homozygous and presence and absence heterozygous samples. In the central panel of (a), one sample (S1603) was expected as presence with a PCR product of present region. However, no PCR product was amplified. (c) The result of PCR for positive control to check the quality of genomic DNA of each sample. (d) Positions of primers for sequence determination of the absent region in LOC107329813. Alignments of 5′ junction sequences and 3′ junction sequences from present and absent alleles are shown in (e) and (f), respectively. (g) Positions of primers for sequence determination of the absent region in LOC10736915–6. (h) Boundaries of absent alleles shown by MinION reads. Figure S3. Combination of presence and absence status of five PAP genes located on one scaffold. Schematic repr [file 12864_2020_6566_MOESM1_ESM.pdf]

Additional file 1

Figure S1

a

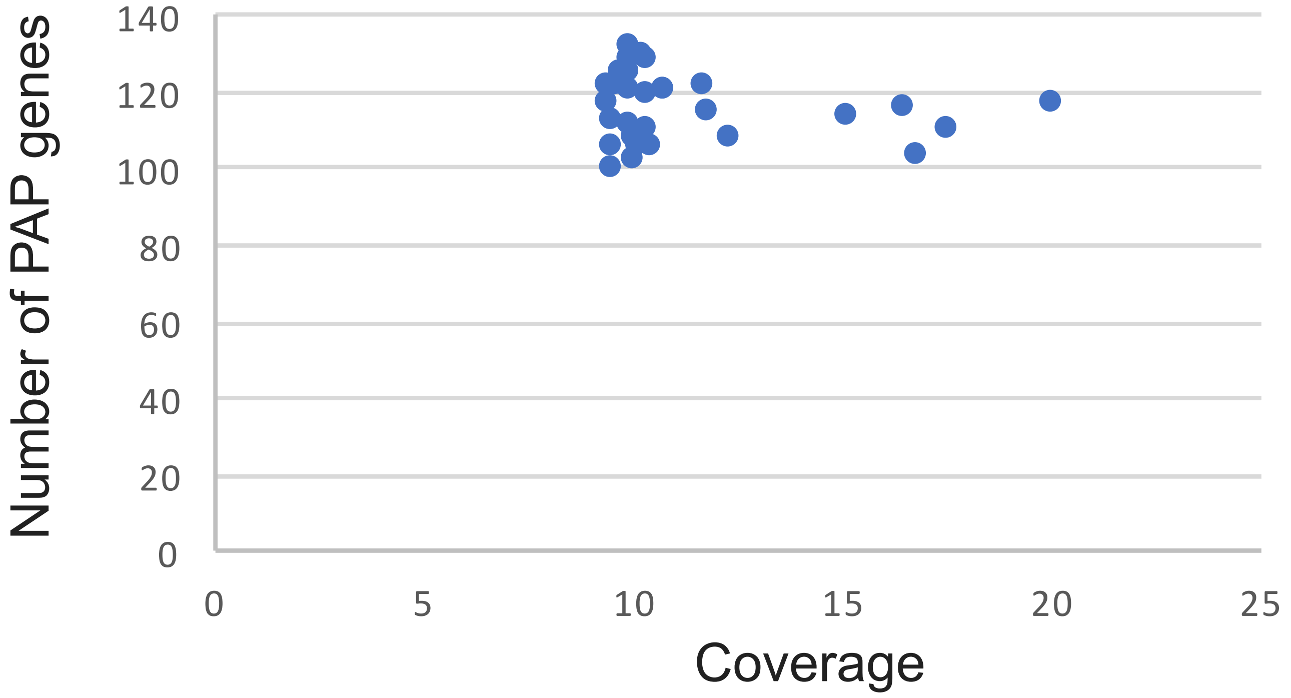

b

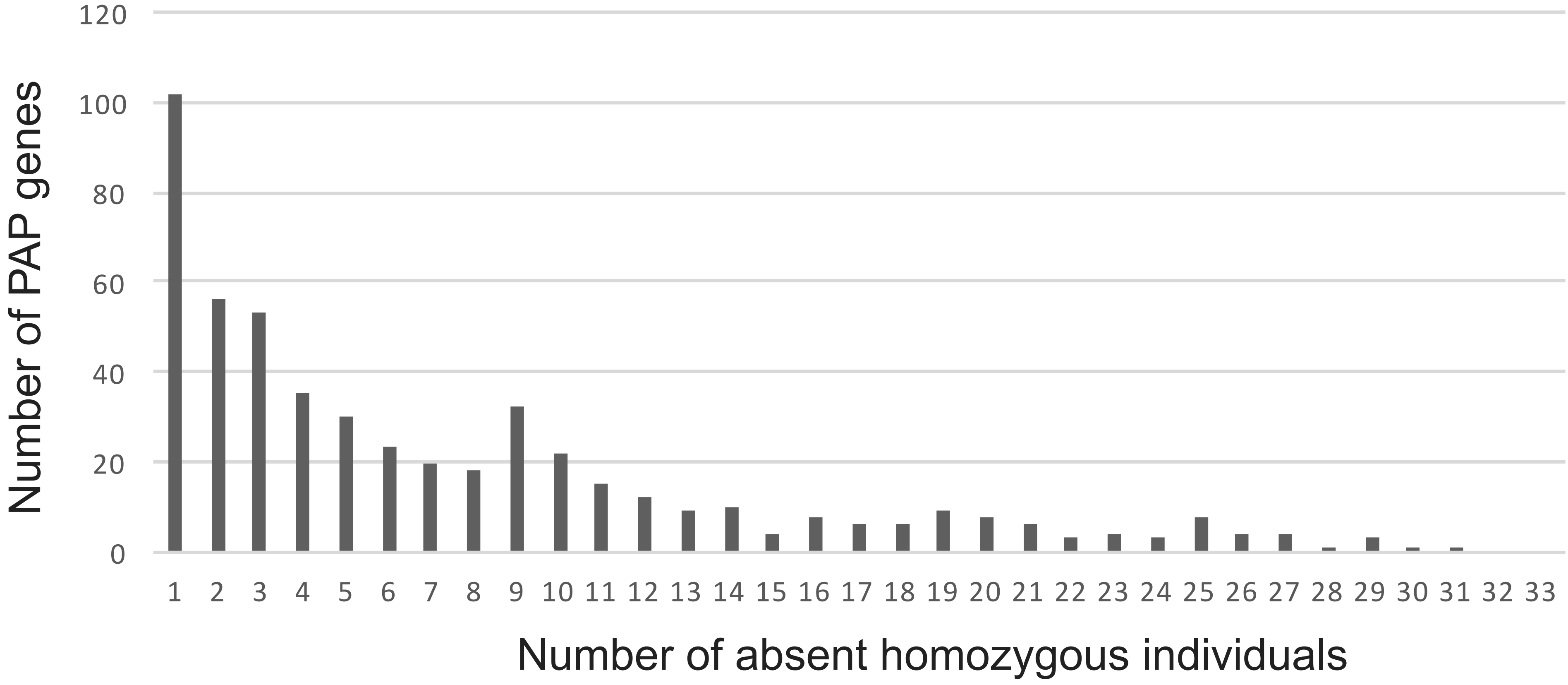

Figure S1. Number of PAP genes in 33 *A. digitifera* individuals
(a) The number of PAP genes (y axis) were plotted against mapping read coverage (x axis) for 33 individuals. (b) The number of PAP genes that were identified as absent homozygous in 1 to 33 individuals. The x-axis represents the numbers of absent homozygous individuals comparing with A. digitifera genome (ver. 1.1) for each PAP gene. The y-axis represents the number of PAP genes. 20% (102) PAP genes appeared in only one individual, and the remaining 80% (n = 414) appeared more than once.

Figure S2

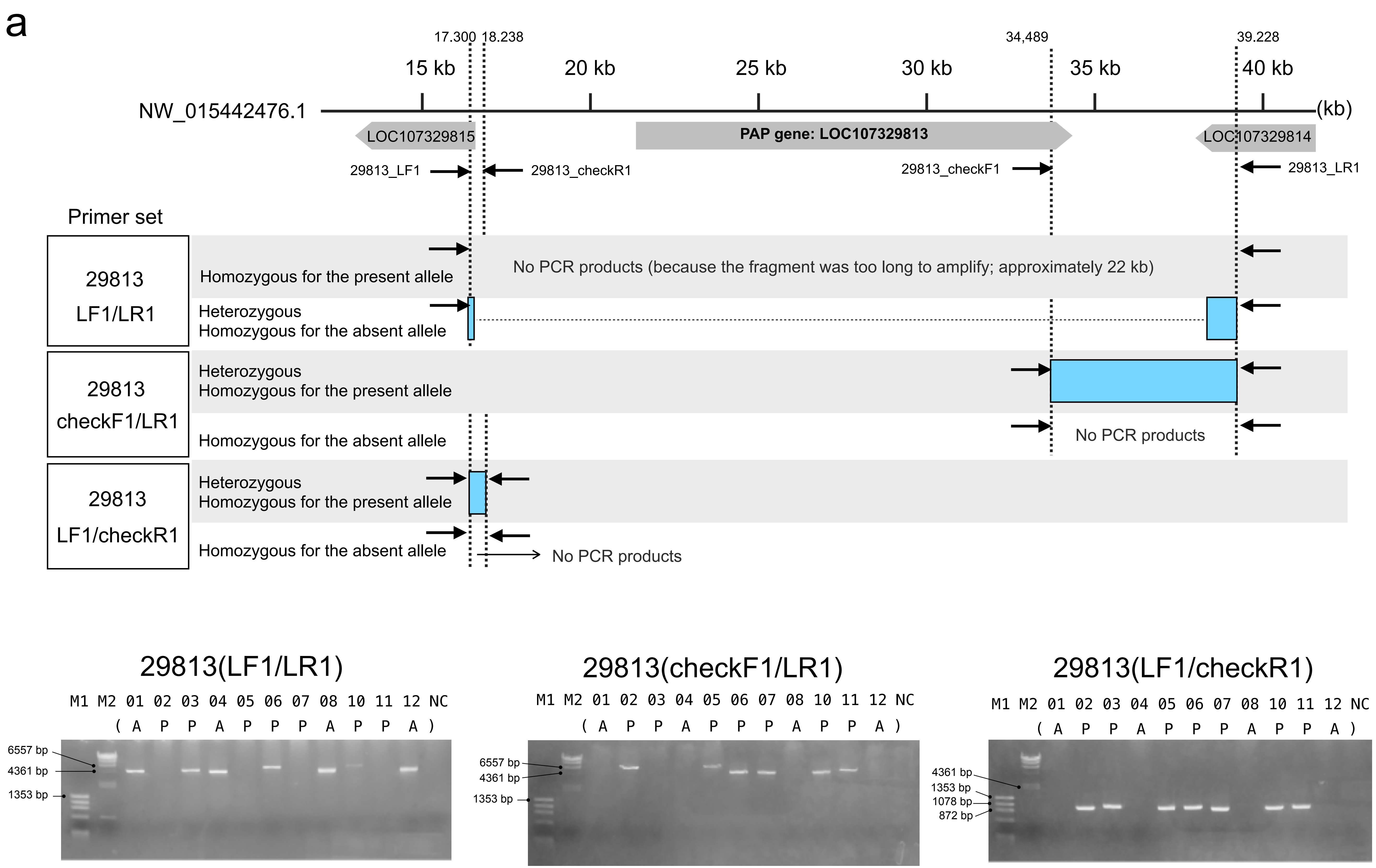

a
17.300
18.238
34,489
39.228
15 kb
20 kb
25 kb
30 kb
35 kb
40 kb
NW_015442476.1
(kb)
LOC107329815
PAP gene: LOC107329813
LOC107329814
29813_LF1
29813_checkR1
29813_checkF1
29813_LR1
Primer set
29813 LF1/LR1
Homozygous for the present allele
No PCR products (because the fragment was too long to amplify; approximately 22 kb)
Heterozygous
Homozygous for the absent allele
29813 checkF1/LR1
Heterozygous
Homozygous for the present allele
Homozygous for the absent allele
No PCR products
29813 LF1/checkR1
Heterozygous
Homozygous for the present allele
Homozygous for the absent allele
No PCR products
29813(LF1/LR1)
M1 M2 01 02 03 04 05 06 07 08 10 11 12 NC
(A P P A P P P A P P A)
6557 bp
4361 bp
1353 bp
29813(checkF1/LR1)
M1 M2 01 02 03 04 05 06 07 08 10 11 12 NC
(A P P A P P P A P P A)
6557 bp
4361 bp
1353 bp
29813(LF1/checkR1)
M1 M2 01 02 03 04 05 06 07 08 10 11 12 NC
(A P P A P P P A P P A)
4361 bp
1353 bp
1078 bp
872 bp

Figure S2

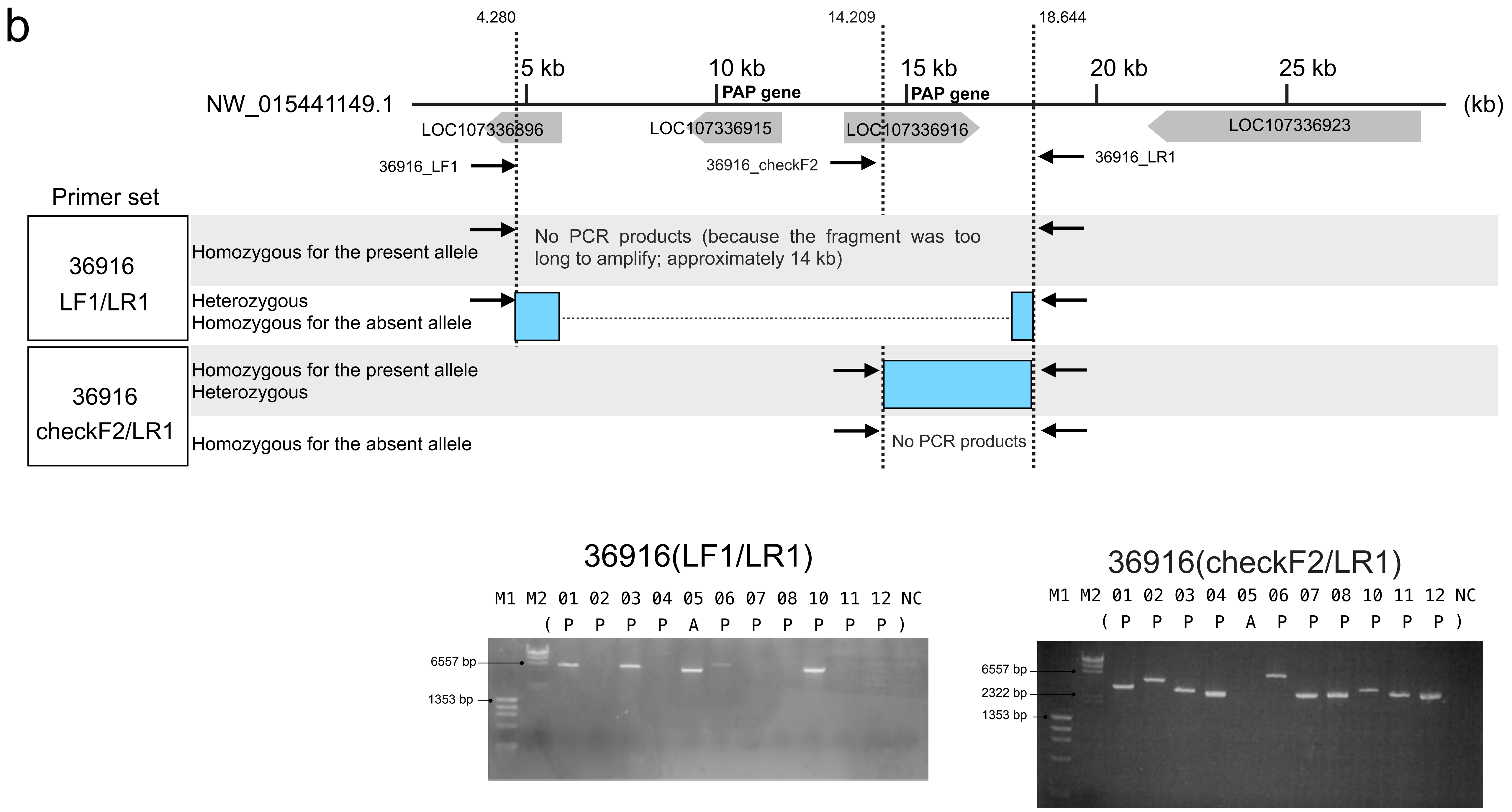
b
4.280
14.209
18.644
5 kb
10 kb
15 kb
20 kb
25 kb
NW_015441149.1
(kb)
PAP gene
PAP gene
LOC107336896
LOC107336915
LOC107336916
LOC107336923
36916_LF1
36916_checkF2
36916_LR1
Primer set
36916
LF1/LR1
Homozygous for the present allele
No PCR products (because the fragment was too long to amplify; approximately 14 kb)
Heterozygous
Homozygous for the absent allele
36916
checkF2/LR1
Homozygous for the present allele
Heterozygous
Homozygous for the absent allele
No PCR products
36916(LF1/LR1)
M1 M2 01 02 03 04 05 06 07 08 10 11 12 NC
(P P P P A P P P P P P)
6557 bp
1353 bp
36916(checkF2/LR1)
M1 M2 01 02 03 04 05 06 07 08 10 11 12 NC
(P P P P A P P P P P P)
6557 bp
2322 bp
1353 bp

Figure S2 (continued)

C

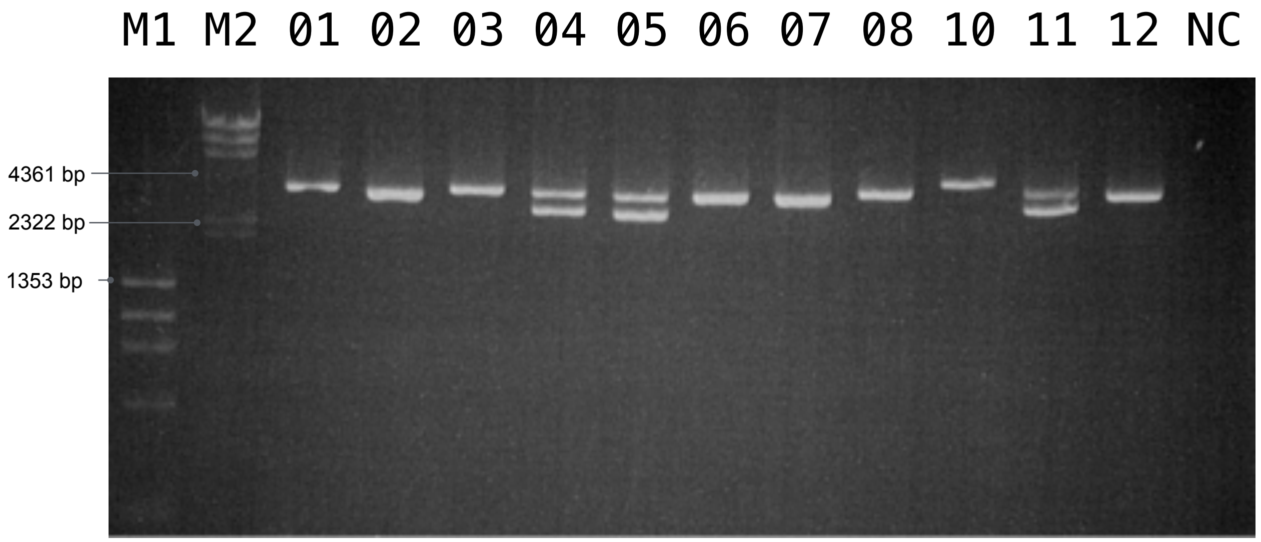

Figure S2 (continued)

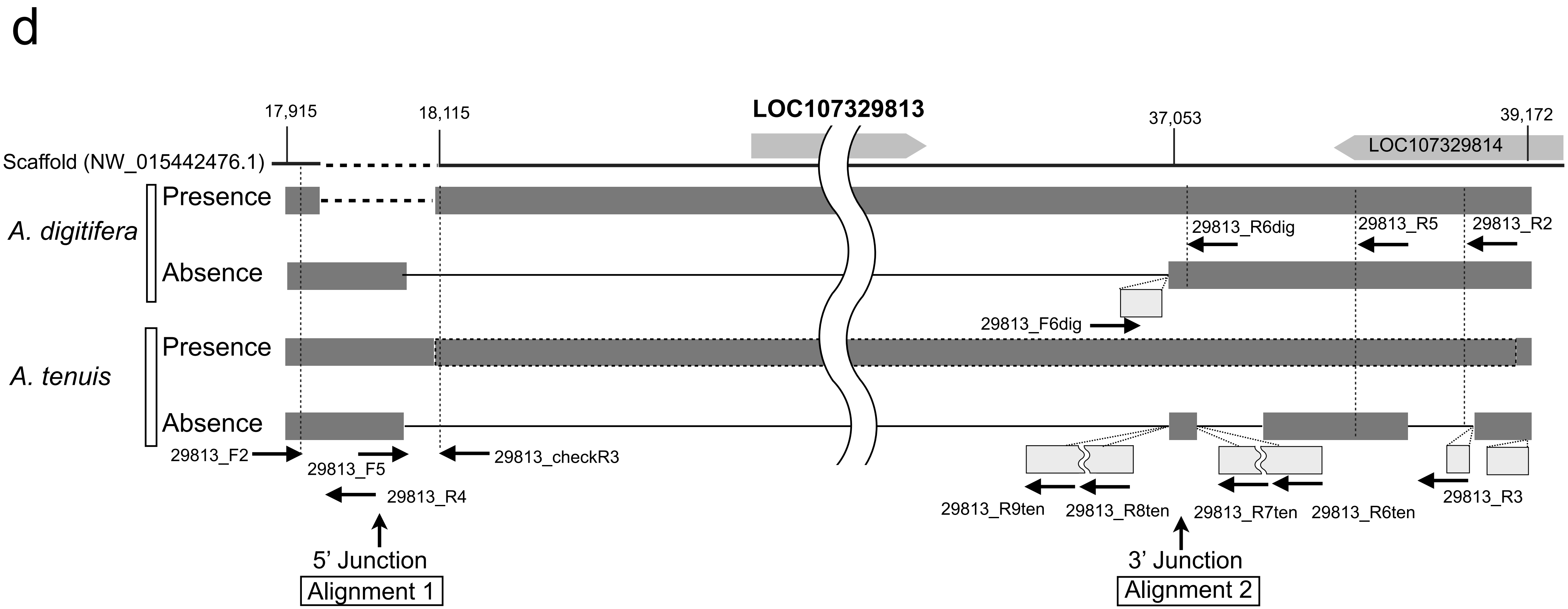

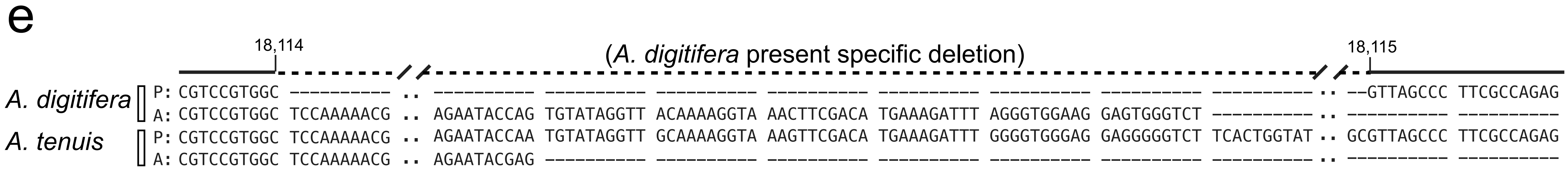

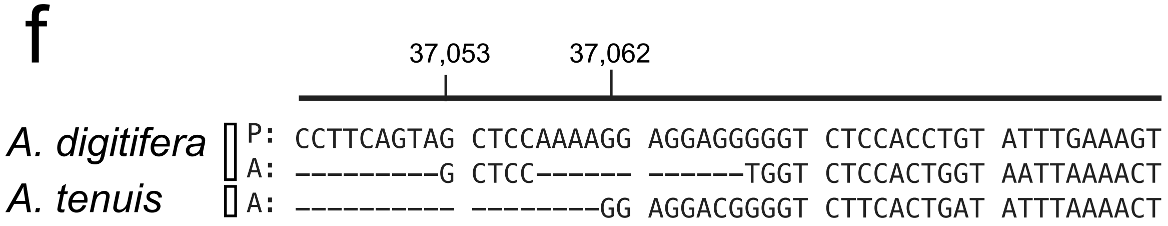

Figure S2 (continued)

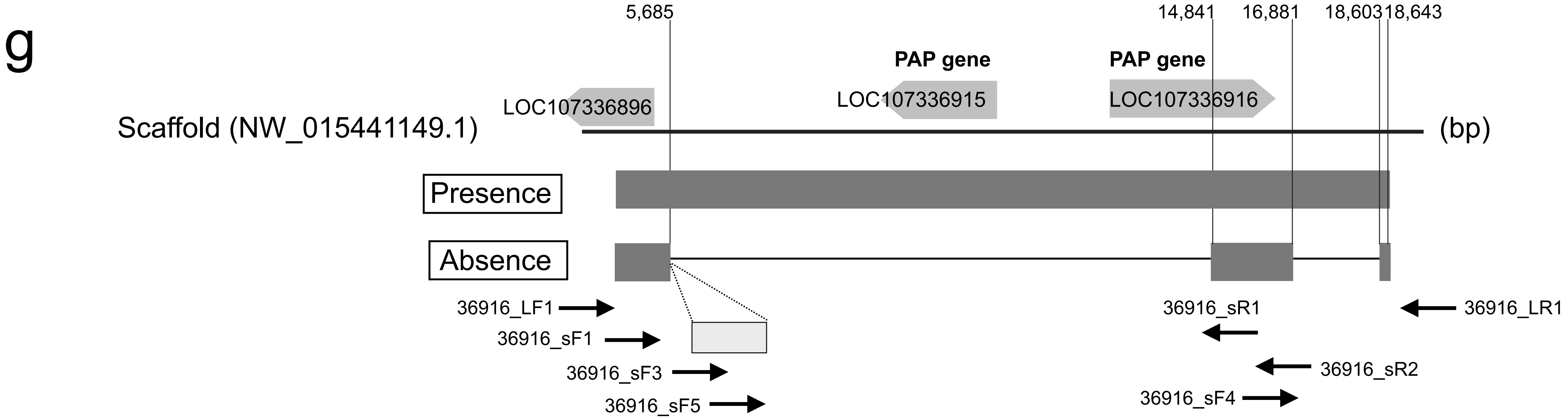

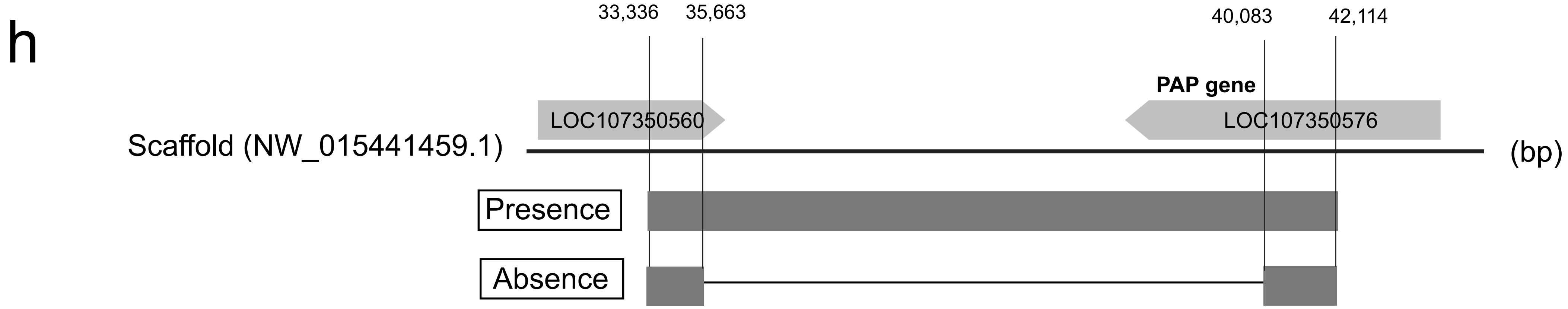

Figure S2. Electrophoresis patterns of PAPs among 11 *A. digitifera* individuals and the structures of PAP loci
The results of PCR products of (a) LOC107329813 and (b) LOC10736915-6. Positions of primers are indicated by arrows, under the schematic representations of genes. Primer names are given under each arrow. M1 and M2 indicate the molecular markers, ϕX174 HaeIII digest and λ-HindIII digest, respectively. Sample IDs S1601-S1612 are indicated as 01-12. NC indicates negative control. The state of presence (P) and absence (A) estimated by the no coverage region are also shown above the photos. Presence state included both presence homozygous and presence and absence heterozygous samples. In the central panel of (a), one sample (S1603) was expected as presence with a PCR product of present region. However, no PCR product was amplified. This may be due to a lack of coverage (indicating deletion) at the primer binding region. (c) The result of PCR for positive control to check the quality of genomic DNA of each sample. (d) Positions of primers for sequence determination of the absent region in LOC107329813. Alignments of 5' junction sequences and 3' junction sequences from present and absent alleles are shown in (e) and (f), respectively. (g) Positions of primers for sequence determination of the absent region in LOC10736915-6. (h) Boundaries of absent alleles shown by MinION reads.

Figure S3

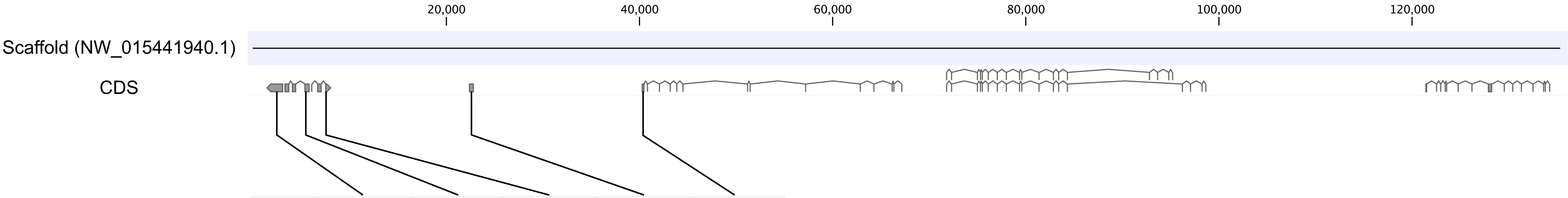

	Sample ID	LOC107326878	LOC107326879	LOC107326880	LOC107326882	LOC107326875
Okinawa	Mz1	P	P	P	P	P
	Od10	P	P	A	P	P
	Od11	P	A	A	P	P
	Od12	P	P	P	P	P
	Od13	A	A	A	A	P
	Od19	A	A	A	P	P
	Od23	P	P	P	P	P
	ohdo1	A	A	P	A	A
	ohdo3	P	A	P	P	P
	S1601	P	P	P	P	P
	S1603	P	P	P	P	P
	S1606	P	P	P	P	P
Kerama	KrA10	P	A	A	A	P
	KrA12	P	P	P	P	P
	KrA13	P	P	P	P	P
	KrA4	A	A	P	P	P
	KrA6	P	P	P	P	P
	KrC10	P	P	P	P	P
	KrC11	P	P	P	P	P
	KrC12	P	P	P	P	P
	KrC13	P	P	P	P	P
	KrC14	P	P	P	P	P
	KrC15	P	P	P	P	P
	KrC5	P	P	P	P	P
	KrC7	P	P	A	P	P
	KrC8	P	P	P	P	P
	KrC9	A	A	A	P	P
	KrE1	P	P	P	P	P
	KrE2	P	A	A	P	P
	KrE4	P	P	P	P	P
	KrE5	P	P	P	P	P
	KrE6	P	P	P	P	P
	KrE8	A	A	A	P	P

P Presence

A Absence

Figure S3. Combination of presence and absence status of eight PAP genes located on one scaffold
Schematic representations of a scaffold (NW_015441940.1) with gene positions (arrows) shown at the top. Rows indicate 33 individuals from two subpopulations. Columns are PAP genes, and the positions are shown by gray arrows at the top. Presence and absence status is shown by white and grey, respectively.

Figure S4

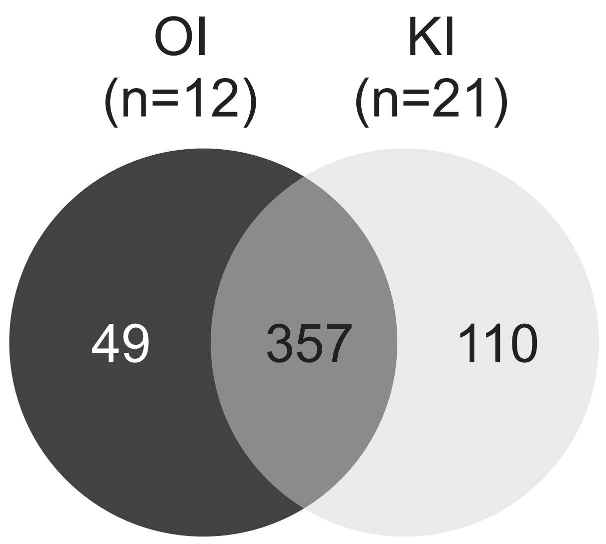

Figure S4. The Venn diagram of PAP genes identified in two subpopulations

The numbers of PAP genes specific to subpopulations and shared among subpopulations are in the Venn diagram.

Figure S5

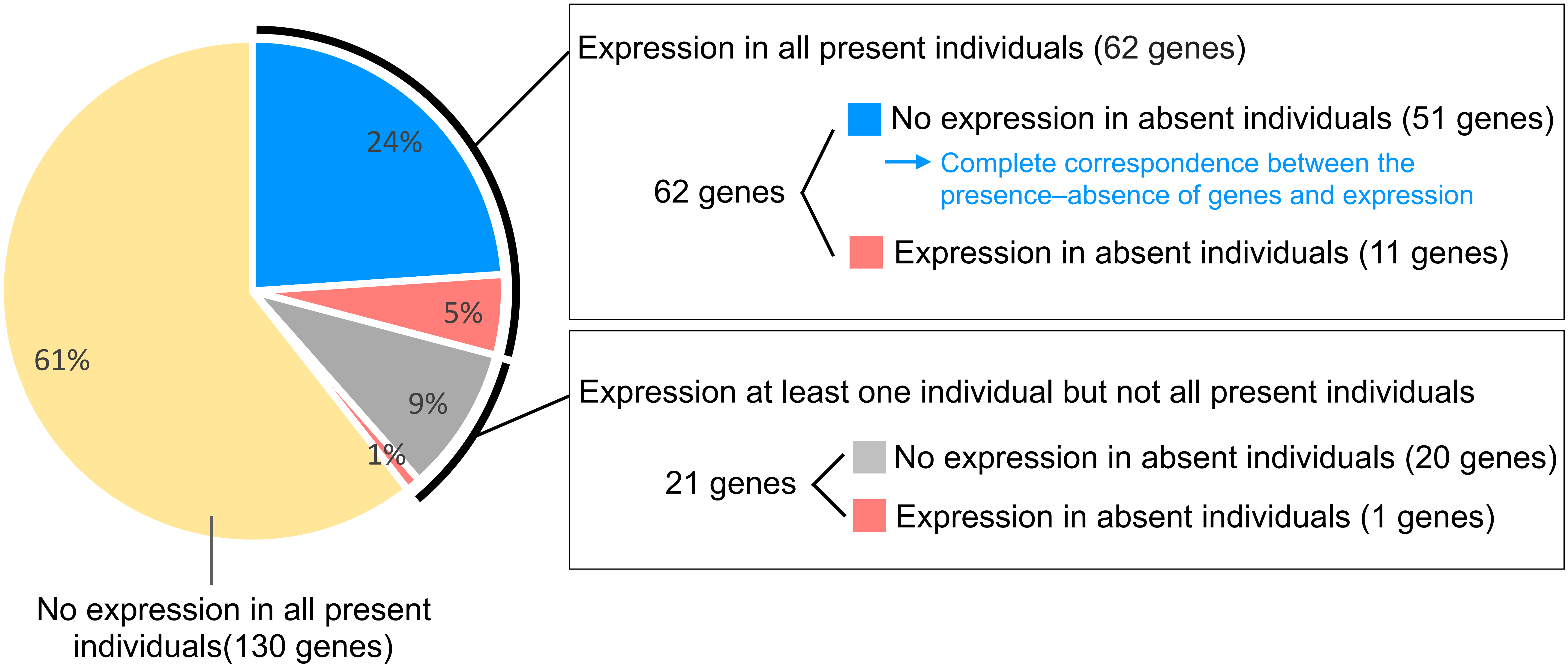

Figure S5. Expressions of genes showing PAPs in three individuals.

Figure S6

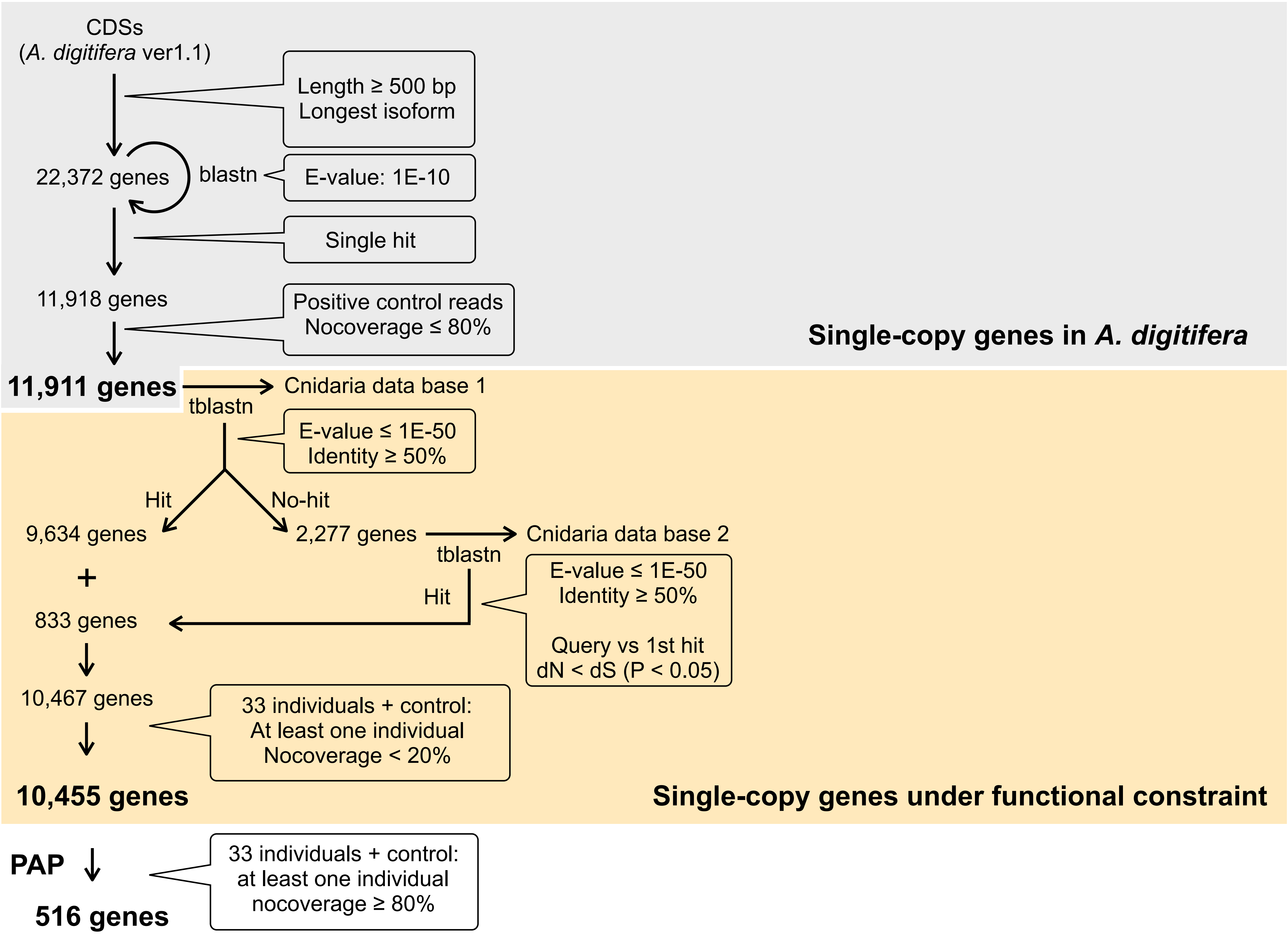

Figure S6. The schematic representation of identification of single-copy genes under functional constraint.

Table S1

Table S1. Overview of high-throughput sequencing

Sample ID	Species	Source	Accession number	Total reads	Mapped reads (%)	Average coverage (CDS paired read only)
S1601	*A. digitifera*	genomic DNA	DRR108003*	91,827,028	68.0	16.9
S1602	*A. digitifera*	genomic DNA	DRR108004*	38,251,050	76.1	6.4
S1603	*A. digitifera*	genomic DNA	DRR108005*	89,613,452	66.3	16.5
S1604	*A. digitifera*	genomic DNA	DRR108006*	43,717,968	76.4	7.3
S1605	*A. digitifera*	genomic DNA	DRR108007*	42,246,308	75.4	6.8
S1606	*A. digitifera*	genomic DNA	DRR108008*	92,762,540	69.1	17.6
S1607	*A. digitifera*	genomic DNA	DRR108009*	36,664,662	76.4	6.3
S1608	*A. digitifera*	genomic DNA	DRR108010*	34,580,790	73.4	5.5
S1610	*A. digitifera*	genomic DNA	DRR108024*	42,615,662	77.2	7.5
S1611	*A. digitifera*	genomic DNA	DRR108011*	40,117,898	76.6	6.7
S1612	*A. digitifera*	genomic DNA	DRR108012*	35,147,552	75.3	5.7
T3	*A. tenuis*	genomic DNA	DRR110778	74,177,804	69.0	19.9
T4	*A. tenuis*	genomic DNA	DRR110779	79,726,028	68.8	21.4
T5	*A. tenuis*	genomic DNA	DRR110780	65,492,982	68.9	17.6
T8	*A. tenuis*	genomic DNA	DRR110781	74,726,820	68.5	20.1
T9	*A. tenuis*	genomic DNA	DRR110782	81,311,224	68.8	21.6
T10	*A. tenuis*	genomic DNA	DRR110783	84,059,950	68.8	22.5
S1601	*A. digitifera*	RNA	DRR108013*	75,591,730	43.5	-
S1603	*A. digitifera*	RNA	DRR108015*	53,761,100	40.1	-
S1606	*A. digitifera*	RNA	DRR108018*	62,627,534	43.7	-
-	*A. digitifera*	RNA	SRX1534820**	97,170,470	81.8	-

*from Takahashi-Kariyazono *et al.* 2018, **from Mohamed AR *et al.* 2016 (see reference)

Table S2

Table S2. Gene IDs and descriptions of PAP genes identified in 33 *A. digitifera*

Gene ID	Description	Expression	TE
LOC107326866	PREDICTED: uncharacterized protein LOC107326866	-	-
LOC107326875	E3ubiquitin-proteinligaseTRAIP-like	Expressed	-
LOC107326878	Werner syndrome ATP-dependent helicase homolog	-	-
LOC107326879	Transposon Tf2-6 poly	Expressed	TE
LOC107326880	PREDICTED: uncharacterized protein LOC107326880	Expressed	-
LOC107326882	PREDICTED: uncharacterized protein LOC107326882	Expressed	-
LOC107326911	uncharacterizedproteinK02A2.6-like	-	-
LOC107326977	ATP-dependent DNA helicase -like	-	-
LOC107327031	RNA-directed DNA polymerase homolog	-	TE
LOC107327207	uncharacterizedproteinLOC107327207	Expressed	-
LOC107327264	PREDICTED: uncharacterized protein LOC107327264	-	-
LOC107327277	BTBandMATHdomain-containingprotein38-like	Expressed	-
LOC107327284	PREDICTED:uncharacterized LOC107327284mRNA	Expressed	-
LOC107327381	PREDICTED: uncharacterized protein LOC107327381	-	-
LOC107327384	PREDICTED: uncharacterized protein LOC107327384	-	-
LOC107327386	ATP-dependent helicase SGS1-like	Expressed	-
LOC107327399	nuclease HARBI1	-	-
LOC107327401	adenosine receptor A2b-like	Expressed	-
LOC107327549	PREDICTED: uncharacterized protein K02A2.6-like	Expressed	-
LOC107327591	nuclease HARBI1	-	-
LOC107327607	coiled-coil domain-containing 82-like	-	-
LOC107327666	nucleoporin GLE1-like	-	-
LOC107327668	PREDICTED: uncharacterized protein LOC107327668	-	-
LOC107327737	Retrovirus-related Pol poly from transposon	-	TE
LOC107327738	sporulation kinase mde3-like [Orbicella faveolata]	-	-
LOC107327743	PREDICTED: uncharacterized protein LOC107327743	-	-
LOC107327916	PREDICTED: uncharacterized protein LOC107327916	-	-
LOC107327920	heat shock 70 kDa 12A-like	Expressed	-
LOC107327921	CAP-Gly domain-containing linker 1-like isoform X2	Expressed	-
LOC107327922	CAP-Gly domain-containing linker 1-like isoform X2	Expressed	-
LOC107328052	Transposable element P transposase	-	TE
LOC107328072	PREDICTED: uncharacterized protein LOC107328072	Expressed	-
LOC107328111	PREDICTED: uncharacterized protein LOC107328111	Expressed	-
LOC107328136	RNA-directed DNA polymerase from mobile element jockey-like	-	TE
LOC107328140	PREDICTED: uncharacterized protein LOC107328140	-	-
LOC107328262	uncharacterizedproteinLOC107328262	-	-
LOC107328282	PREDICTED: uncharacterized protein LOC107328282	-	-
LOC107328296	myb X	Expressed	-
LOC107328301	PREDICTED: uncharacterized protein LOC107328301	-	-
LOC107328359	receptor-type tyrosine- phosphatase F-like isoform X18	-	-
LOC107328442	PREDICTED: uncharacterized protein K02A2.6-like	-	-
LOC107328474	PREDICTED: uncharacterized protein LOC107328474	-	-
LOC107328478	PREDICTED: uncharacterized protein LOC107328478	-	-
LOC107328498	PREDICTED: LOW QUALITY PROTEIN: trichohyalin-like	-	-
LOC107328547	PREDICTED: uncharacterized protein LOC107328547	-	-
LOC107328549	PREDICTED: uncharacterized protein LOC107328549	-	-
LOC107328551	PREDICTED: uncharacterized protein LOC107328551	-	-
LOC107328574	PREDICTED: uncharacterized protein LOC107328574	-	-
LOC107328613	uncharacterizedproteinLOC107328613	Expressed	-
LOC107328615*	venom prothrombin activator pseutarin-C non-catalytic subunit-like isoform X1 [Orbicella faveolata]	-	-
LOC107328665	nuclease HARBI1	-	-
LOC107328693	PREDICTED: uncharacterized protein LOC107328693	Expressed	-
LOC107328703	ATP-dependent DNA helicase -like	-	-
LOC107328777	ATP-dependent DNA helicase PIF1	-	-
LOC107328778	PREDICTED: uncharacterized protein LOC107328778	-	-
LOC107328831	nuclease HARBI1	-	-
LOC107328899*	DELTA-alicitoxin-Pse2b-like [Orbicella faveolata]	Expressed	-
LOC107328915	PREDICTED: uncharacterized protein LOC107328915 isoform X1	-	-
LOC107328985	roundabout homolog 1-like	Expressed	-
LOC107329014	uncharacterizedproteinLOC107329014isoformX1	-	-
LOC107329017	PREDICTED: uncharacterized protein LOC107329017	Expressed	-
LOC107329092	acid phosphatase type 7-like	Expressed	-
LOC107329116	PREDICTED: uncharacterized protein LOC107329116	-	-
LOC107329124	PREDICTED: uncharacterized protein LOC107329124	Expressed	-
LOC107329127	tiggertransposableelement-derivedprotein4-like	-	TE
LOC107329135	PREDICTED: uncharacterized protein LOC107329135	Expressed	-
LOC107329217	PREDICTED: uncharacterized protein LOC107329217	Expressed	-
LOC107329253	RNA-directed DNA polymerase from mobile element jockey-like	-	TE
LOC107329270	PREDICTED: uncharacterized protein LOC107329270	Expressed	-
LOC107329282	bromodomain adjacent to zinc finger domain 2B-like	Expressed	-
LOC107329369	probable RNA-dependent RNA polymerase 1	-	-
LOC107329412	PREDICTED: uncharacterized protein LOC107329412	Expressed	-
LOC107329498	nuclease HARBI1	-	-
LOC107329567	PREDICTED: uncharacterized protein LOC107329567	Expressed	-
LOC107329587	PREDICTED: uncharacterized protein LOC107329587	-	-
LOC107329677	fibroblast growth factor receptor homolog 1-like	Expressed	-
LOC107329697	uncharacterizedproteinLOC107329697	Expressed	-
LOC107329738	PREDICTED: uncharacterized protein LOC107329738	Expressed	-
LOC107329778	PREDICTED: uncharacterized protein LOC107329778	Expressed	-
LOC107329779	PREDICTED: uncharacterized protein LOC107329779	-	-
LOC107329813	PREDICTED: uncharacterized protein LOC107329813	Expressed	-
LOC107329830	PREDICTED: uncharacterized protein LOC107329830	Expressed	-
LOC107329831	PREDICTED: uncharacterized protein LOC107329831	Expressed	-
LOC107329867	PREDICTED: uncharacterized protein K02A2.6-like	-	-
LOC107329883	piggyBac transposable element-derived 4-like	-	TE
LOC107329926	centrosomal of 89 kDa-like	Expressed	-
LOC107329943	Inhibitor of growth 3	-	-
LOC107329960	isovaleryl- mitochondrial	Expressed	-
LOC107329961	ATP-dependent DNA helicase PIF1	-	-
LOC107329971	craniofacial development 2-like	-	-
LOC107330022	zinc finger 862-like	Expressed	-
LOC107330150	PREDICTED: LOW QUALITY PROTEIN: uncharacterized protein LOC107330150, partial	-	-
LOC107330260	PREDICTED: uncharacterized protein LOC107330260	Expressed	-
LOC107330321	PREDICTED: uncharacterized protein LOC107330321 isoform X1	Expressed	-

Table S2 (continued)

Gene ID	Description	Expression	TE
LOC107330360	PREDICTED: uncharacterized protein LOC107330360	-	-
LOC107330433	growth hormone secretagogue receptor type 1-like	Expressed	-
LOC107330437	Cytosolic non-specific dipeptidase	Expressed	-
LOC107330455	disintegrin and metallo ase domain-containing 15-like	Expressed	-
LOC107330533	PREDICTED: uncharacterized protein LOC107330533	-	-
LOC107330537	ATP-dependent DNA helicase PIF1-like	-	-
LOC107330545	arf-GAP with coiled- ANK repeat and PH domain-containing 2-	Expressed	-
LOC107330582	nuclease HARBI1	Expressed	-
LOC107330631	Kinesin light chain	-	-
LOC107330707	uncharacterized kDa in hlyA 3 region-like	Expressed	-
LOC107330713	lecithin retinol acyltransferase family	Expressed	-
LOC107330780	PREDICTED: uncharacterized protein LOC107330780	-	-
LOC107330786	zinc finger MYM-type 1-like	-	-
LOC107330925	substance-P receptor-like [Orbicella faveolata]	Expressed	-
LOC107331191	PREDICTED: uncharacterized protein LOC107331191	Expressed	-
LOC107331279	uncharacterizedproteinLOC107331279	Expressed	-
LOC107331400	PREDICTED: uncharacterized protein LOC107331400	-	-
LOC107331438	nuclease HARBI1	Expressed	-
LOC107331603	PREDICTED: uncharacterized protein LOC107331603 isoform X1	Expressed	-
LOC107331790	PREDICTED: uncharacterized protein LOC107331790	Expressed	-
LOC107331818	uncharacterizedproteinLOC107331818	-	-
LOC107331871	uncharacterizedproteinLOC107331871	Expressed	-
LOC107332035	PREDICTED: uncharacterized protein LOC107332035	Expressed	-
LOC107332059	sporulation kinase mde3-like [Orbicella faveolata]	-	-
LOC107332154	PREDICTED: uncharacterized protein LOC107332154	Expressed	-
LOC107332253	piggyBac transposable element-derived 4-like	-	TE
LOC107332262	uncharacterizedproteinLOC107332262	Expressed	-
LOC107332275	PREDICTED: uncharacterized protein LOC107332275 isoform X1	-	-
LOC107332276	PREDICTED: uncharacterized protein LOC107332276	Expressed	-
LOC107332277	THAP domain-containing 2	-	-
LOC107332295	PREDICTED: uncharacterized protein LOC107332295	-	-
LOC107332428	neurogenic locus notch homolog 2-like	Expressed	-
LOC107332449	PREDICTED: uncharacterized protein LOC107332449 isoform X1	-	-
LOC107332451	PREDICTED: uncharacterized protein LOC107332451	Expressed	-
LOC107332493	low-density lipo receptor-related 4-like isoform X1 [Orbicella faveolata]	Expressed	-
LOC107332543	synaptonemal complex 2-like	-	-
LOC107332563	dyneinheavychain7 2Caxonemal-like	-	-
LOC107332687	PREDICTED: uncharacterized protein LOC107332687	-	-
LOC107332709	FAR1-RELATED SEQUENCE 7	Expressed	-
LOC107332772	basic proline-rich -like	-	-
LOC107332832	PREDICTED: uncharacterized protein LOC107332832	-	-
LOC107332848	PREDICTED: uncharacterized protein LOC107332848	Expressed	-
LOC107333229	PREDICTED: uncharacterized protein LOC107333229 isoform X1	Expressed	-
LOC107333440	ubiquitin-ribosomal 60S subunit L40B fusion	Expressed	-
LOC107333546	exonuclease R569	Expressed	-
LOC107333553	PREDICTED: uncharacterized protein LOC107333553	-	-
LOC107333568	ankyrin repeat	Expressed	-
LOC107333805	PREDICTED: uncharacterized protein LOC107333805	-	-
LOC107334028	mab-21-like 3	Expressed	-
LOC107334104	PREDICTED: LOW QUALITY PROTEIN: uncharacterized protein LOC107334104	-	-
LOC107334110	nuclease HARBI1	-	-
LOC107334120	PREDICTED: uncharacterized protein LOC107334120	-	-
LOC107334122	nuclease HARBI1	-	-
LOC107334256	aspartyl asparaginyl beta-hydroxylase-like	Expressed	-
LOC107334257	FAD-linked oxidoreductase DDB_G0289697-like [Orbicella faveolata]	Expressed	-
LOC107334258	PREDICTED: uncharacterized protein LOC107334258	Expressed	-
LOC107334388	neuropeptide FF receptor 2-like isoform X1	Expressed	-
LOC107334677	PREDICTED: uncharacterized protein LOC107334677	-	-
LOC107334724	PREDICTED: uncharacterized protein LOC107334724	Expressed	-
LOC107334853	choline dehydrogenase	Expressed	-
LOC107334884	PREDICTED: uncharacterized protein LOC107334884	-	-
LOC107334901	cholesterol oxidase-like isoform X2	Expressed	-
LOC107334940	PREDICTED: uncharacterized protein LOC107334940	-	-
LOC107335089	PREDICTED: uncharacterized protein LOC107335089	Expressed	-
LOC107335101	RNA-directed DNA polymerase homolog	-	TE
LOC107335105	RNA-directed DNA polymerase from mobile element jockey-like	-	TE
LOC107335135	ATP-dependent DNA helicase PIF7-like	-	-
LOC107335145	PREDICTED: uncharacterized protein LOC107335145 isoform X1	Expressed	-
LOC107335263	uncharacterizedproteinLOC107335263	-	-
LOC107335266	Retrovirus-related Pol poly from transposon TNT 1-94	-	TE
LOC107335269	PREDICTED: uncharacterized protein LOC107335269	Expressed	-
LOC107335681	PREDICTED: uncharacterized protein LOC107335681	Expressed	-
LOC107335928	zinc finger 397-like	-	-
LOC107336168	uncharacterizedproteinLOC107336168	-	-
LOC107336333	PREDICTED: uncharacterized protein LOC107336333	-	-
LOC107336339	collagen alpha-1(VII) chain-like	Expressed	-
LOC107336429	G2 M phase-specific E3 ubiquitin- ligase-like	-	-
LOC107336430	G2 M phase-specific E3 ubiquitin- ligase-like	-	-
LOC107336432	PREDICTED: uncharacterized protein LOC107336432	-	-
LOC107336581	transmembrane matrix receptor MUP-4-like	-	-
LOC107336621	nuclease HARBI1	-	-
LOC107336631	PREDICTED: uncharacterized protein LOC107336631	-	-
LOC107336737	PREDICTED: uncharacterized protein LOC107336737	-	-
LOC107336915	guanylate-binding 5-	Expressed	-
LOC107336916	stress response NST1-like	Expressed	-
LOC107336931	skeletalasparticacid-richprotein1-likeisoformX1	Expressed	-
LOC107337209	PREDICTED: uncharacterized protein LOC107337209	-	-
LOC107337338	PREDICTED: uncharacterized protein LOC107337338	Expressed	-
LOC107337384	tetratricopeptide repeat 28-like	Expressed	-
LOC107337721	Integrase recombinase xerD-	-	-
LOC107337834	PREDICTED: uncharacterized protein LOC107337834	-	-
LOC107337889	glomulin-likeisoformX1	Expressed	-
LOC107338030	PREDICTED: uncharacterized protein K02A2.6-like	-	-
LOC107338045	uncharacterizedproteinLOC107338045	-	-
LOC107338067	peptide-N(4)-(N-acetyl-beta-glucosaminyl)asparagine amidase-like isoform X2	Expressed	-
LOC107338192	ras-related Rab-1A	Expressed	-

Table S2 (continued)

Gene ID	Description	Expression	TE
LOC107338236	PREDICTED: uncharacterized protein LOC107338236	-	-
LOC107338274	PREDICTED: uncharacterized protein LOC107338274	Expressed	-
LOC107338306	PREDICTED: uncharacterized protein LOC107338306	Expressed	-
LOC107338342	PREDICTED: uncharacterized protein LOC107338342	-	-
LOC107338483	PREDICTED: LOW QUALITY PROTEIN: uncharacterized protein LOC107338483	-	-
LOC107338516	PREDICTED: uncharacterized protein LOC107338516	Expressed	-
LOC107338524	PREDICTED: uncharacterized protein LOC107338524	Expressed	-
LOC107338582	steroid receptor RNA activator 1-like	Expressed	-
LOC107338602	RNA-directed DNA polymerase	-	TE
LOC107338605	PREDICTED: uncharacterized protein LOC107338605	-	-
LOC107338638	CD63 antigen-like	Expressed	-
LOC107338652	uncharacterizedproteinLOC107338652	Expressed	-
LOC107338654	PREDICTED: uncharacterized protein K02A2.6-like	Expressed	-
LOC107338670	TNF receptor-associated factor 5-like	Expressed	-
LOC107338680	PREDICTED: tyrosinase-like	Expressed	-
LOC107338684	ANTAGONIST OF LIKE HETEROCHROMATIN PROTEIN 1-like	-	-
LOC107338701	PREDICTED: LOW QUALITY PROTEIN: uncharacterized protein LOC107338701	-	-
LOC107338713	PREDICTED: uncharacterized protein LOC107338713	Expressed	-
LOC107338752	CGG triplet repeat-binding 1	-	-
LOC107338878	PREDICTED: uncharacterized protein LOC107338878	Expressed	-
LOC107338885	sodium-dependent phosphate transport 2B-like	Expressed	-
LOC107339264	PREDICTED: uncharacterized protein LOC107339264	-	-
LOC107339283	ATP-dependent DNA helicase PIF1	-	-
LOC107339284	ATP-dependent DNA helicase PIF1	Expressed	-
LOC107339341	Stage V sporulation K	-	-
LOC107339442	microfibril-associated glyco 4-like isoform X1	Expressed	-
LOC107339630	RNA-directed DNA polymerase from mobile element jockey-like	-	TE
LOC107339661	PREDICTED: LOW QUALITY PROTEIN: uncharacterized protein LOC107339661	Expressed	-
LOC107339671	calcium-dependent kinase 1-like	Expressed	-
LOC107339686	G2 M phase-specific E3 ubiquitin- ligase	Expressed	-
LOC107339752	alpha-amylase 2-like	Expressed	-
LOC107339909	PREDICTED: uncharacterized protein LOC107339909	-	-
LOC107339967	PREDICTED: uncharacterized protein LOC107339967	-	-
LOC107340000	PREDICTED: uncharacterized protein LOC107340000	-	-
LOC107340010	PREDICTED: uncharacterized protein LOC107340010	-	-
LOC107340011	PREDICTED: uncharacterized protein LOC107340011	-	-
LOC107340150	PREDICTED: uncharacterized protein LOC107340150	-	-
LOC107340242	PREDICTED: uncharacterized protein LOC107340242	Expressed	-
LOC107340281	polyubiquitin 8-like	Expressed	-
LOC107340308	PREDICTED: uncharacterized protein LOC107340308	-	-
LOC107340343	PREDICTED: uncharacterized protein LOC107340343	Expressed	-
LOC107340344	PREDICTED: uncharacterized protein LOC107340344	-	-
LOC107340345	PREDICTED: uncharacterized protein LOC107340345 isoform X1	Expressed	-
LOC107340388	PREDICTED: uncharacterized protein LOC107340388	Expressed	-
LOC107340631	PREDICTED: uncharacterized protein LOC107340631	-	-
LOC107340632	PREDICTED: uncharacterized protein LOC107340632	-	-
LOC107340680	RNA-directed DNA polymerase from transposon X-element	-	TE
LOC107340732	fibroblast growth factor receptor homolog 1-like	Expressed	-
LOC107340786	tiggertransposableelement-derivedprotein6-like	-	TE
LOC107340856	PREDICTED: uncharacterized protein LOC107340856	Expressed	-
LOC107341130	fibropellin-1-like isoform X2	Expressed	-
LOC107341260	PREDICTED: uncharacterized protein LOC107341260	Expressed	-
LOC107341356	RNA-directed DNA polymerase from mobile element jockey	-	TE
LOC107341426	PREDICTED: uncharacterized protein LOC107341426	Expressed	-
LOC107341702	PREDICTED: uncharacterized protein LOC107341702	-	-
LOC107341741	PREDICTED: uncharacterized protein LOC107341741	-	-
LOC107341742	uncharacterizedproteinLOC107341742	Expressed	-
LOC107341785	PREDICTED: uncharacterized protein LOC107341785	Expressed	-
LOC107341895	uncharacterizedproteinLOC107341895	Expressed	-
LOC107341939	transient receptor potential cation channel subfamily M member 6-like	-	-
LOC107341940	PREDICTED: uncharacterized protein LOC107341940	-	-
LOC107342004	PREDICTED: uncharacterized protein LOC107342004	-	-
LOC107342055	ATP-dependent DNA helicase	Expressed	-
LOC107342088	P2X purinoceptor 7-like	Expressed	-
LOC107342111	PREDICTED: uncharacterized protein LOC107342111	Expressed	-
LOC107342128	PREDICTED: uncharacterized protein LOC107342128	Expressed	-
LOC107342140	von Willebrand factor D and EGF domain-containing -like	Expressed	-
LOC107342212	EF-hand calcium-binding domain-containing 11-like	Expressed	-
LOC107342327	PREDICTED: uncharacterized protein LOC107342327	Expressed	-
LOC107342364	calponin homology domain-containing DDB_G0272472-like	-	-
LOC107342607	PREDICTED: uncharacterized protein LOC107342607	Expressed	-
LOC107342650	PREDICTED: uncharacterized protein LOC107342650	-	-
LOC107342652	PREDICTED: uncharacterized protein LOC107342652	Expressed	-
LOC107342667	PREDICTED: uncharacterized protein LOC107342667	-	-
LOC107342698	E3 ubiquitin- ligase TRIM71-like	Expressed	-
LOC107342908	PREDICTED: uncharacterized protein LOC107342908 isoform X1	Expressed	-
LOC107342909	nuclease HARBI1	Expressed	-
LOC107343191	unc-13 homolog C-like	-	-
LOC107343213	PREDICTED: uncharacterized protein LOC107343213	-	-
LOC107343217	PREDICTED: uncharacterized protein LOC107343217	-	-
LOC107343293	PREDICTED: uncharacterized protein LOC107343293	-	-
LOC107343318	uncharacterizedproteinLOC107343318	Expressed	-
LOC107343320	52 kDa repressor of the inhibitor of the kinase-like	Expressed	-
LOC107343351	calcitonin receptor-like	Expressed	-
LOC107343414	X-prolyl aminopeptidase	Expressed	-
LOC107343550	PREDICTED: uncharacterized protein LOC107343550	Expressed	-
LOC107343551	PREDICTED: uncharacterized protein LOC107343551 isoform X1	Expressed	-
LOC107343583	PREDICTED: uncharacterized protein LOC107343583 isoform X1	Expressed	-
LOC107343786	PREDICTED: uncharacterized protein LOC107343786	Expressed	-
LOC107344006	zinc finger and SCAN domain-containing 29-like	-	-
LOC107344088	PREDICTED: mucin-like protein	Expressed	-
LOC107344292	PREDICTED: uncharacterized protein LOC107344292	-	-
LOC107344307	uncharacterizedproteinLOC107344307	Expressed	-
LOC107344312	PREDICTED: uncharacterized protein LOC107344312	Expressed	-
LOC107344361	PREDICTED: uncharacterized protein LOC107344361	-	-
LOC107344365	rasGEF domain-containing serine threonine- kinase X-like	Expressed	-

Table S2 (continued)

Gene ID	Description	Expression	TE
LOC107344473	E3 ubiquitin- ligase RNF213-like	-	-
LOC107344506	serine-rich adhesin for platelets-like	Expressed	-
LOC107344534	RNA polymerase II-associated 3-like	Expressed	TE
LOC107344544	ATP-binding cassette sub-family B member mitochondrial	-	-
LOC107344698	RNA-directed DNA polymerase from transposon BS	-	TE
LOC107344717	PREDICTED: uncharacterized protein LOC107344717	Expressed	-
LOC107344722	PREDICTED: uncharacterized protein K02A2.6-like	-	-
LOC107344723	PREDICTED: uncharacterized protein LOC107344723	-	-
LOC107344948	sterile alpha motif domain-containing 9-like	-	-
LOC107345000	RNA-directed DNA polymerase from mobile element jockey-like	-	TE
LOC107345218	piggyBac transposable element-derived 4-like	-	TE
LOC107345281	translationally-controlled tumor homolog	Expressed	-
LOC107345363	von Willebrand factor A domain-containing 7-like	Expressed	-
LOC107345370	probable Werner syndrome ATP-dependent helicase homolog 1	Expressed	-
LOC107345388	PREDICTED: uncharacterized protein LOC107345388	Expressed	-
LOC107345518	N-lysine methyltransferase METTL21A-like	Expressed	-
LOC107345595	PREDICTED: uncharacterized protein LOC107345595	-	-
LOC107345652	PREDICTED: uncharacterized protein LOC107345652	Expressed	-
LOC107345686	PREDICTED: uncharacterized protein LOC107345686	Expressed	-
LOC107345821	PREDICTED: uncharacterized protein LOC107345821	Expressed	-
LOC107345859	PREDICTED: uncharacterized protein LOC107345859	-	-
LOC107345953	PREDICTED: uncharacterized protein LOC107345953	-	-
LOC107345954	RNA-directed DNA polymerase from transposon BS	-	TE
LOC107346009	PREDICTED: uncharacterized protein LOC107346009	Expressed	-
LOC107346079	uncharacterizedproteinLOC107346079	-	-
LOC107346195	uncharacterizedproteinK02A2.6-like	-	-
LOC107346200	PREDICTED: uncharacterized protein LOC107346200	-	-
LOC107346201	Retrovirus-related Pol poly from transposon	-	TE
LOC107346222	PREDICTED: uncharacterized protein LOC107346222	Expressed	-
LOC107346245	PREDICTED: uncharacterized protein LOC107346245	Expressed	-
LOC107346322	PREDICTED: uncharacterized protein LOC107346322	Expressed	-
LOC107346547	PREDICTED: uncharacterized protein LOC107346547	Expressed	-
LOC107346734	Transposon Ty3-I Gag-Pol poly	-	TE
LOC107346746	PREDICTED: uncharacterized protein LOC107346746	-	-
LOC107346750	PREDICTED: uncharacterized protein LOC107346750	-	-
LOC107346751	G2 M phase-specific E3 ubiquitin- ligase-like	-	-
LOC107346839	PREDICTED: uncharacterized protein LOC107346839	Expressed	-
LOC107346953	myb X	Expressed	-
LOC107346968	General transcription factor II-I repeat domain-containing 1	Expressed	-
LOC107347038	PREDICTED: uncharacterized protein LOC107347038	Expressed	-
LOC107347039	nuclease HARBI1	Expressed	-
LOC107347148	PREDICTED: uncharacterized protein LOC107347148	-	-
LOC107347149	PREDICTED: uncharacterized protein LOC107347149	Expressed	-
LOC107347179*	endothelin-converting enzyme 1-like	Expressed	-
LOC107347338	integrase recombinase xerD homolog	Expressed	-
LOC107347354	PREDICTED: uncharacterized protein LOC107347354	-	-
LOC107347890	uncharacterizedproteinLOC107347890	Expressed	-
LOC107347931	poly [ADP-ribose] polymerase 14-like	Expressed	-
LOC107347932	poly [ADP-ribose] polymerase 14-like	Expressed	-
LOC107348010	PREDICTED: uncharacterized protein LOC107348010	Expressed	-
LOC107348058	PREDICTED: uncharacterized protein LOC107348058	Expressed	-
LOC107348266	ganglioside GM2 activator-like	Expressed	-
LOC107348297	PREDICTED: uncharacterized protein LOC107348297 isoform X1	Expressed	-
LOC107348302	PREDICTED: uncharacterized protein LOC107348302	-	-
LOC107348336	uncharacterizedproteinLOC107348336	-	-
LOC107348470	THAP domain-containing 6	-	-
LOC107348471	PREDICTED: uncharacterized protein LOC107348471	-	-
LOC107348486	PREDICTED: uncharacterized protein LOC107348486	Expressed	-
LOC107348580	PREDICTED: uncharacterized protein LOC107348580	-	-
LOC107348583	PREDICTED: uncharacterized protein LOC107348583	-	-
LOC107348597	PREDICTED: uncharacterized protein LOC107348597	Expressed	-
LOC107348605	uncharacterizedproteinLOC107348605isoformX1	Expressed	-
LOC107348755	PREDICTED: uncharacterized protein K02A2.6-like	-	-
LOC107348965	integrase recombinase xerD homolog	-	-
LOC107348998	PREDICTED: uncharacterized protein LOC107348998	-	-
LOC107349000	PREDICTED: uncharacterized protein LOC107349000	-	-
LOC107349002	PREDICTED: uncharacterized protein LOC107349002	-	-
LOC107349040	PREDICTED: uncharacterized protein LOC107349040	-	-
LOC107349067	zinc finger MYM-type 3-like	-	-
LOC107349106	BTB POZ domain-containing 6-like	-	-
LOC107349373	PREDICTED: uncharacterized protein LOC107349373	Expressed	-
LOC107349462	PREDICTED: uncharacterized protein LOC107349462	Expressed	-
LOC107349606	PREDICTED: uncharacterized protein LOC107349606 isoform X1	-	-
LOC107349624	PREDICTED: uncharacterized protein LOC107349624	Expressed	-
LOC107349625	PREDICTED: uncharacterized protein LOC107349625	-	-
LOC107349633	PREDICTED: uncharacterized protein LOC107349633	-	-
LOC107349901	nuclease HARBI1	Expressed	-
LOC107349932	PREDICTED: uncharacterized protein LOC107349932	Expressed	-
LOC107349944	PREDICTED: uncharacterized protein LOC107349944	Expressed	-
LOC107350219	MAC perforin- and kringle-domains-containing	Expressed	-
LOC107350267	golgin subfamily A member 6 2 isoform X2	Expressed	-
LOC107350319	PREDICTED: uncharacterized protein LOC107350319	-	-
LOC107350332	PREDICTED: uncharacterized protein LOC107350332	-	-
LOC107350363	PREDICTED: uncharacterized protein LOC107350363	Expressed	-
LOC107350462	PREDICTED: uncharacterized protein LOC107350462	-	-
LOC107350576	PREDICTED: uncharacterized protein LOC107350576 isoform X1	Expressed	-
LOC107350578	piggyBac transposable element-derived 4-like	-	TE
LOC107350624	PREDICTED: uncharacterized protein LOC107350624	Expressed	-
LOC107350719	PREDICTED: uncharacterized protein LOC107350719	Expressed	-
LOC107350727	nuclease HARBI1	-	-
LOC107350804	ALP1-like isoform X1	-	-
LOC107350863	PREDICTED: uncharacterized protein LOC107350863	Expressed	-
LOC107350864	PREDICTED: uncharacterized protein LOC107350864	-	-
LOC107350865	PREDICTED: uncharacterized protein LOC107350865	-	-
LOC107350914	F-box only 43-like	Expressed	-
LOC107351010	PREDICTED: uncharacterized protein LOC107351010	Expressed	-

Table S2 (continued)

Gene ID	Description	Expression	TE
LOC107351183	PREDICTED: uncharacterized protein LOC107351183	-	-
LOC107351184	PREDICTED: uncharacterized protein LOC107351184	-	-
LOC107351207	PREDICTED: uncharacterized protein LOC107351207 isoform X1	Expressed	-
LOC107351285	RNA-directed DNA polymerase from mobile element jockey	-	TE
LOC107351746	Pro-Pol poly	Expressed	-
LOC107351786	Pro-Pol poly	Expressed	-
LOC107351788	Ubiquitin-60S ribosomal L40	Expressed	-
LOC107351916	PREDICTED: uncharacterized protein LOC107351916	-	-
LOC107351959	retrograde of 51 kDa-like	Expressed	-
LOC107352034	stress response NST1-like	-	-
LOC107352036	PREDICTED: uncharacterized protein LOC107352036	Expressed	-
LOC107352062	PREDICTED: uncharacterized protein LOC107352062	Expressed	-
LOC107352063	---NA---	Expressed	-
LOC107352110	histidine N-acetyltransferase-like	Expressed	-
LOC107352251	PREDICTED: uncharacterized protein LOC107352251	Expressed	-
LOC107352371	inner centromere -like	-	-
LOC107352373	PREDICTED: uncharacterized protein LOC107352373	-	-
LOC107352513	PREDICTED: uncharacterized protein LOC107352513	Expressed	-
LOC107352632	uncharacterizedproteinLOC107352632	-	-
LOC107352797	PREDICTED: uncharacterized protein LOC107352797	-	-
LOC107352927	uncharacterizedproteinLOC107352927	Expressed	-
LOC107353055	piggyBac transposable element-derived 4-like	Expressed	TE
LOC107353109	Pre-mRNA-splicing factor ATP-dependent RNA helicase PRP16	Expressed	-
LOC107353121	PREDICTED: uncharacterized protein LOC107353121	Expressed	-
LOC107353157	ATP-dependent DNA helicase Q1	-	-
LOC107353220	PREDICTED: uncharacterized protein LOC107353220 isoform X1	Expressed	-
LOC107353261	PREDICTED: uncharacterized protein LOC107353261, partial	Expressed	-
LOC107353276	PREDICTED: uncharacterized protein LOC107353276	Expressed	-
LOC107353325	annexin A13-like	-	-
LOC107353455	vesicular integral-membrane VIP36-like	Expressed	-
LOC107353553	phthioceranic hydroxyphthioceranic acid synthase-like isoform X2	Expressed	-
LOC107353587	PREDICTED: uncharacterized protein LOC107353587	Expressed	-
LOC107353610	cyclin-dependent kinase 2-like	-	-
LOC107353678	ATP-dependent DNA helicase PIF1-like	-	-
LOC107353712	PREDICTED: uncharacterized protein LOC107353712	-	-
LOC107353718	PREDICTED: uncharacterized protein LOC107353718	-	-
LOC107353739	PREDICTED: uncharacterized protein LOC107353739	Expressed	-
LOC107353740	PREDICTED: uncharacterized protein LOC107353740	Expressed	-
LOC107353741	PREDICTED: uncharacterized protein LOC107353741	-	-
LOC107353795	piggyBactransposableelement-derivedprotein4-like	-	TE
LOC107353874	uncharacterizedproteinDDB_G0271670-likeisoformX1	Expressed	-
LOC107353937	Pol poly	-	-
LOC107353948	PREDICTED: uncharacterized protein LOC107353948	Expressed	-
LOC107354018	PREDICTED: uncharacterized protein LOC107354018	Expressed	-
LOC107354169	PREDICTED: uncharacterized protein LOC107354169	-	-
LOC107354264	PREDICTED: uncharacterized protein LOC107354264	-	-
LOC107354266	PREDICTED: uncharacterized protein LOC107354266	-	-
LOC107354268	nuclease HARBI1	-	-
LOC107354271	ATP-dependent DNA helicase Q1	-	-
LOC107354272	PREDICTED: uncharacterized protein LOC107354272	-	-
LOC107354457	PREDICTED: protein MB21D2-like	Expressed	-
LOC107354504	PREDICTED: uncharacterized protein LOC107354504	-	-
LOC107354529	acyl-coenzyme A synthetase mitochondrial-like	Expressed	-
LOC107354532	jerky homolog-like	-	-
LOC107354746	PREDICTED: uncharacterized protein LOC107354746	Expressed	-
LOC107354754	L-gulonolactone oxidase-like	Expressed	-
LOC107354964	HMG domain-containing 3-like	-	-
LOC107354970	PREDICTED: uncharacterized protein LOC107354970	-	-
LOC107355034	PREDICTED: uncharacterized protein K02A2.6-like	-	-
LOC107355065	phosphatidylinositol-glycan-specific phospholipase D-like	Expressed	-
LOC107355068	uncharacterizedproteinLOC107355068	Expressed	-
LOC107355137	PREDICTED: uncharacterized protein LOC107355137 isoform X1	Expressed	-
LOC107355265	Retrovirus-related Pol poly from transposon opus	-	TE
LOC107355310	uncharacterizedproteinLOC107355310	-	-
LOC107355313	PREDICTED: uncharacterized protein LOC107355313	-	-
LOC107355382	PREDICTED: uncharacterized protein LOC107355382	-	-
LOC107355433	PREDICTED: uncharacterized protein LOC107355433	-	-
LOC107355458	PREDICTED: uncharacterized protein LOC107355458	-	-
LOC107355530	THAP domain-containing 1	Expressed	-
LOC107355532	LIGHT-DEPENDENT SHORT HYPOCOTYLS 6	-	-
LOC107355545	PREDICTED: uncharacterized protein LOC107355545	Expressed	-
LOC107355551	craniofacial development 2-like	-	-
LOC107355612	bark beetle-like isoform X2 [Orbicella faveolata]	Expressed	-
LOC107355636	F-box only 36-like	Expressed	-
LOC107355646	uncharacterizedproteinK02A2.6-like	-	-
LOC107355650	zinc finger 862-like	Expressed	-
LOC107355654	PREDICTED: uncharacterized protein LOC107355654	Expressed	-
LOC107355801	PREDICTED: uncharacterized protein LOC107355801 isoform X1	Expressed	-
LOC107355908	PREDICTED: uncharacterized protein K02A2.6-like	Expressed	-
LOC107356019	fibronectin type III domain-containing -like	Expressed	-
LOC107356048	Integrase recombinase xerD-like	Expressed	-
LOC107356137	nuclease HARBI1	-	-
LOC107356237	PREDICTED: uncharacterized protein LOC107356237	-	-
LOC107356238	PREDICTED: uncharacterized protein LOC107356238	Expressed	-
LOC107356262	RNA-directed DNA polymerase from mobile element jockey-like	-	TE
LOC107356328	PREDICTED: uncharacterized protein LOC107356328	Expressed	-
LOC107356355	unc-13 homolog C-like	-	-
LOC107356421	PREDICTED: uncharacterized protein LOC107356421	Expressed	-
LOC107356427	Transposon Tf2-9 poly	-	TE
LOC107356440	reverse [Rhodotorula toruloides NP11	-	TE
LOC107356492	PREDICTED: uncharacterized protein LOC107356492, partial	-	-
LOC107356511	PREDICTED: uncharacterized protein LOC107356511	-	-
LOC107356513	PREDICTED: uncharacterized protein LOC107356513	-	-
LOC107356514	PREDICTED: uncharacterized protein LOC107356514	-	-
LOC107356544	PREDICTED: uncharacterized protein LOC107356544	Expressed	-
LOC107356569	PREDICTED: uncharacterized protein LOC107356569	-	-

Table S2 (continued)

Gene ID	Description	Expression	TE
LOC107356691	nuclease HARBI1	-	-
LOC107356743	PREDICTED: uncharacterized protein LOC107356743	-	-
LOC107356754	PREDICTED: uncharacterized protein LOC107356754	Expressed	-
LOC107356783	PREDICTED: uncharacterized protein LOC107356783	-	-
LOC107357027	PREDICTED: uncharacterized protein K02A2.6-like	-	-
LOC107357102	PREDICTED: uncharacterized protein LOC107357102	Expressed	-
LOC107357107	PREDICTED: uncharacterized protein LOC107357107	-	-
LOC107357238	OTU domain-containing 3-like	-	-
LOC107357335	PREDICTED: uncharacterized protein LOC107357335	Expressed	-
LOC107357336	PREDICTED: uncharacterized protein LOC107357336	Expressed	-
LOC107357459	PREDICTED: uncharacterized protein LOC107357459	-	-
LOC107357580	PREDICTED: uncharacterized protein LOC107357580	-	-
LOC107357584	PREDICTED: uncharacterized protein LOC107357584	-	-
LOC107357664	PREDICTED: uncharacterized protein LOC107357664	-	-
LOC107357668	PREDICTED: uncharacterized protein LOC107357668	-	-
LOC107357726	tropomyosin beta chain-like	Expressed	-
LOC107357727	myosin heavy chain-like isoform X2	Expressed	-
LOC107357779	nuclease HARBI1	-	-
LOC107357781	PREDICTED: uncharacterized protein LOC107357781 isoform X1	-	-
LOC107357807	PREDICTED: uncharacterized protein LOC107357807	-	-
LOC107357852	PREDICTED: uncharacterized protein LOC107357852	-	-
LOC107358056	uncharacterizedproteinLOC107358056	-	-
LOC107358094	PREDICTED: uncharacterized protein LOC107358094	-	-
LOC107358154	PREDICTED: uncharacterized protein LOC107358154	Expressed	-
LOC107358215	PREDICTED: uncharacterized protein LOC107358215	-	-
LOC107358216	low-density lipo receptor-related 5-like	Expressed	-
LOC107358302	PREDICTED: uncharacterized protein LOC107358302	Expressed	-
LOC107358346	PREDICTED: uncharacterized protein LOC107358346	-	-
LOC107358477	vicilin-like seed storage At2g18540	Expressed	-
LOC107358520	fukutin-related -like	Expressed	-
LOC107358527	PREDICTED: uncharacterized protein LOC107358527	-	-
LOC107358645	mab-21-like 2	Expressed	-
LOC107358656*	Toxin -60A	Expressed	-
LOC107358728	uncharacterizedproteinK02A2.6-like	-	-
LOC107358734	uncharacterizedproteinLOC107358734	-	-
LOC107358841	PREDICTED: uncharacterized protein K02A2.6-like	-	-
LOC107358885	PREDICTED: uncharacterized protein LOC107358885	-	-
LOC107358907	PREDICTED: uncharacterized protein LOC107358907 isoform X1	-	-

Table S3

Table S3. Percentage of no coverage region and RPKM of 51 PAP genes

Gene	No coverage (%)			RPKM		
	S1601	S1603	S1606	S1601	S1603	S1606
LOC107340281	0	100	0	42	0	97
LOC107341130	100	0	100	0	48	0
LOC107338192	0	100	0	44	0	42
LOC107339671	100	100	0	0	0	41
LOC107332276	100	100	8	0	0	39
LOC107353121	0	0	99	27	19	0
LOC107340242	89	0	0	0	5	26
LOC107354746	0	100	0	15	0	22
LOC107329813	100	0	0	0	19	6
LOC107334853	100	0	100	0	19	0
LOC107333229	0	86	86	19	0	0
LOC107358216	100	0	0	0	17	17
LOC107329778	10	100	100	17	0	0
LOC107353740	0	0	100	14	16	0
LOC107339442	100	0	100	0	13	0
LOC107335681	0	100	1	3	0	12
LOC107329567	0	0	100	12	10	0
LOC107343583	100	0	100	0	10	0
LOC107354754	0	0	100	4	9	0
LOC107332428	91	0	0	0	9	5
LOC107337384	0	100	0	8	0	2
LOC107329017	0	0	89	7	5	0
LOC107353553	100	0	0	0	6	3
LOC107353109	2	100	100	6	0	0
LOC107354457	0	100	0	3	0	6
LOC107343786	0	9	93	1	6	0
LOC107338878	100	2	2	0	5	1
LOC107353276	2	100	0	5	0	3
LOC107342607	100	0	100	0	5	0
LOC107356421	100	0	0	0	4	4
LOC107357727	100	0	0	0	4	2
LOC107332493	100	0	3	0	1	4
LOC107347932	0	0	93	3	2	0
LOC107350219	100	0	27	0	3	1
LOC107334901	100	0	100	0	3	0
LOC107357726	100	0	0	0	1	2
LOC107351788	0	100	86	2	0	0
LOC107353220	71	1	100	1	2	0
LOC107355801	100	0	0	0	2	1
LOC107351746	100	8	9	0	2	1
LOC107357102	100	4	100	0	2	0
LOC107351786	100	0	0	0	2	1
LOC107328899	0	0	100	2	1	0
LOC107346222	0	100	100	1	0	0
LOC107355612	99	0	98	0	1	0
LOC107342327	74	97	98	1	0	0
LOC107335145	100	87	0	0	0	1
LOC107342140	20	100	100	1	0	0
LOC107354529	97	0	97	0	1	0
LOC107344506	100	100	0	0	0	1
LOC107358154	100	0	96	0	1	0

Table S4

Table S4. Description and no coverage (%) of newly-identified single-copy genes

ID	Description	No coverage (%)																															
		Mz1	Od10	Od11	Od12	Od13	Od19	Od23	ohdo1	ohdo3	S1601	S1603	S1606	KrA4	KrA6	KrA10	KrA12	KrA13	KrC5	KrC7	KrC8	KrC9	KrC10	KrC11	KrC12	KrC13	KrC14	KrC15	KrE1	KrE2	KrE4	KrE5	KrE6	KrE8
TCONS_00000262.p1_S1603	PREDICTED: uncharacterized protein LOC107358902 [Acropora digitifera]	100	100	100	100	100	100	100	100	100	100	13	100	51	100	100	100	100	43	100	100	100	100	82	100	38	100	100	100	43	100	100	100	100
TCONS_00000350.p1_S1601	hypothetical protein AWC38_SpisGene2967 [Stylophora pistillata]	100	14	11	11	24	100	14	4	0	0	10	0	10	33	2	23	25	25	10	9	16	0	19	15	13	34	24	5	9	0	50	11	0
TCONS_00000674.p1_S1603	PREDICTED: uncharacterized protein K02A2.6-like [Acropora digitifera]	100	100	100	100	100	100	0	0	100	100	0	0	100	100	100	100	100	100	100	100	100	100	100	100	100	100	100	0	100	100	100	100	100
TCONS_00000743.p1_S1601	PREDICTED: uncharacterized protein LOC107338878 [Acropora digitifera]	100	100	100	1	100	0	57	100	100	0	74	1	100	100	7	100	100	100	1	100	100	12	100	100	100	6	65	100	89	9	100	53	100
TCONS_00000747.p1_S1606	uncharacterized protein LOC110041186 [Orbicella faveolata]	7	3	0	3	0	100	54	4	100	5	0	69	100	9	52	6	70	100	100	11	59	23	100	100	0	52	52	100	27	1	22	4	2
TCONS_00000756.p1_S1603	uncharacterized protein LOC110061929 [Orbicella faveolata]	0	100	80	0	0	1	0	0	0	75	0	0	0	0	0	0	0	0	0	28	0	0	0	0	100	0	0	100	0	0	100	100	0
TCONS_00001376.p1_S1606	uncharacterized protein LOC110052694 [Orbicella faveolata]	0	0	0	0	100	0	0	1	100	0	7	0	0	100	0	0	88	100	0	1	1	0	88	0	0	88	100	100	94	100	88	0	0
TCONS_00001577.p1_S1603	PREDICTED: uncharacterized protein LOC107350322 [Acropora digitifera]	22	100	100	100	100	7	100	100	83	100	0	0	100	100	100	100	100	100	0	100	100	100	100	100	100	100	100	100	100	100	100	100	100
TCONS_00001650.p1_S1601	PREDICTED: serine/threonine-protein kinase/endoribonuclease IRE1-like [Cyprinus carpio]	13	100	0	100	0	0	100	2	0	0	0	0	100	0	0	0	100	0	100	47	100	31	40	25	0	100	0	0	86	0	0	100	0
TCONS_00001928.p1_S1601	uncharacterized protein LOC110047120 [Orbicella faveolata]	0	0	0	0	0	0	0	0	100	0	100	100	1	1	1	1	0	100	100	100	1	1	100	0	0	0	100	1	0	0	100	100	0
TCONS_00002187.p1_S1601	Transposon Tf2-8 polyprotein [Exaiptasia pallida]	0	0	0	0	0	0	0	3	0	0	0	0	0	0	0	33	0	0	0	0	0	1	0	0	2	32	3	0	0	6	24	0	0
TCONS_00002213.p1_S1606	hypothetical protein [Pseudomonas soli]	100	100	100	100	100	100	100	100	100	100	0	0	100	100	100	100	100	100	100	100	100	100	100	3	100	0	100	100	100	100	100	100	100
TCONS_00002315.p1_S1601	hypothetical protein AWC38_SpisGene3943 [Stylophora pistillata]	100	100	100	100	100	1	100	0	100	0	100	100	3	100	100	100	100	100	100	100	0	7	100	100	0	100	100	100	100	100	34	0	100
TCONS_00002559.p1_S1601	PREDICTED: uncharacterized protein LOC107357975 [Acropora digitifera]	4	0	6	14	0	0	0	10	1	0	0	0	4	3	14	0	0	70	0	2	0	0	100	13	10	5	33	0	0	6	79	0	8
TCONS_00002939.p1_S1606	uncharacterized protein LOC111105687 [Crassostrea virginica]	0	0	100	100	0	100	100	0	0	0	0	0	0	0	100	0	0	0	100	0	0	0	0	0	100	0	0	0	0	0	0	0	0
TCONS_00003373.p1_S1603	uncharacterized protein LOC110443942 [Mizuhopecten yessoensis]	71	6	100	100	8	7	14	2	1	25	5	10	15	2	30	16	15	4	100	6	8	100	7	5	41	75	29	6	67	17	100	100	3
TCONS_00003838.p1_S1606	hypothetical protein AWC38_SpisGene6108 [Stylophora pistillata]	100	6	100	0	100	100	100	92	1	87	100	1	0	100	100	0	90	100	100	100	100	100	100	0	100	3	100	100	4	100	100	90	100
TCONS_00004422.p1_S1601	uncharacterized protein LOC110056080 [Orbicella faveolata]	0	0	0	0	100	0	0	0	1	0	0	100	0	0	5	3	0	0	0	100	1	100	0	100	0	0	0	100	0	100	100	0	100
TCONS_00004515.p1_S1601	predicted protein [Nematostella vectensis]	7	15	100	13	9	100	100	18	0	0	100	8	7	100	6	7	11	100	5	8	9	0	13	100	11	9	100	100	100	1	2	7	9
TCONS_00004978.p1_S1601	uncharacterized protein LOC110046888 [Orbicella faveolata]	100	81	24	82	100	82	100	100	100	0	100	100	100	0	75	0	0	100	82	100	100	100	100	99	100	77	29	100	100	100	100	100	77
TCONS_00005117.p1_S1603	uncharacterized protein LOC110457385 isoform X1 [Mizuhopecten yessoensis]	6	100	100	100	100	0	100	100	0	100	0	100	100	100	100	0	100	0	100	100	0	100	100	0	0	100	85	0	4	100	100	0	100
TCONS_00005149.p1_S1601	uncharacterized protein LOC110253720 [Exaiptasia pallida]	100	100	100	0	0	100	100	0	100	0	100	100	100	100	100	100	100	100	100	0	0	100	100	100	100	100	100	100	100	100	100	100	0
TCONS_00005236.p1_S1601	hypothetical protein AWC38_SpisGene16958 [Stylophora pistillata]	0	0	0	13	0	0	100	100	0	0	100	100	0	0	0	0	3	0	43	2	0	100	13	0	62	0	0	5	100	0	0	0	0
TCONS_00006396.p1_S1603	guanylate-binding protein 5-like, partial [Orbicella faveolata]	15	13	15	17	100	4	0	14	100	2	0	13	14	100	15	11	100	100	14	0	0	18	100	14	14	100	100	100	100	100	100	13	14
TCONS_00006671.p1_S1603	ParA family protein [Synechococcus sp. RS9917]	100	0	1	4	2	100	100	2	8	1	0	100	100	0	83	1	100	82	5	5	13	0	100	0	0	100	2	83	1	100	82	100	3
TCONS_00006764.p1_S1606	uncharacterized protein LOC110041336 isoform X1 [Orbicella faveolata]	3	0	0	1	100	0	100	0	7	100	0	0	0	29	0	9	82	100	0	100	0	0	1	0	0	0	100	0	0	100	9	0	100
TCONS_00006957.p1_S1606	uncharacterized protein K02A2.6-like [Mizuhopecten yessoensis]	0	0	48	100	0	100	3	64	100	100	100	0	100	100	1	69	8	70	88	100	47	0	48	2	58	100	0	100	56	100	100	100	100
TCONS_00007288.p1_S1601	myosin-7B-like [Orbicella faveolata]	100	100	100	100	100	100	100	100	3	0	100	100	100	100	100	100	100	3	100	100	89	100	1	100	100	100	1	100	0	100	0	0	100
TCONS_00007528.p1_S1601	uncharacterized protein LOC110048798 isoform X1 [Orbicella faveolata]	89	0	86	0	100	100	100	0	1	0	0	100	100	100	100	100	100	95	100	100	2	100	100	3	100	100	5	100	1	100	100	100	93
TCONS_00008035.p1_S1606	PREDICTED: von Willebrand factor D and EGF domain-containing protein-like isoform X1 [Acropora digitifera]	0	0	0	3	100	100	100	0	0	0	0	0	80	1	0	0	0	0	100	100	100	3	1	0	100	100	0	100	100	0	24	100	0
TCONS_00008242.p1_S1606	hypothetical protein AKAW_07882 [Aspergillus kawachii IFO 4308]	100	0	0	100	0	0	0	100	0	0	0	0	0	100	0	0	100	0	5	1	5	100	0	100	100	100	0	1	0	100	100	100	100
TCONS_00008799.p1_S1601	uncharacterized protein LOC110057961 [Orbicella faveolata]	100	100	100	21	100	100	9	100	100	9	100	9	5	24	100	100	10	4	3	100	100	100	100	14	100	3	100	100	0	0	100	100	100
TCONS_00008895.p1_S1601	uncharacterized protein LOC111325754 [Stylophora pistillata]	100	100	100	0	100	90	100	100	100	0	0	100	0	100	100	100	100	100	100	100	100	0	0	100	0	95	100	100	100	100	100	0	2
TCONS_00009726.p1_S1606	Apolipoprotein L3 [Stylophora pistillata]	2	0	88	0	10	88	9	3	6	100	5	0	100	0	18	100	100	0	33	0	0	68	100	5	0	0	5	100	0	6	100	6	100
TCONS_00009962.p1_S1603	hypothetical protein AWC38_SpisGene4407 [Stylophora pistillata]	17	58	62	20	38	32	33	45	43	56	41	67	39	50	38	42	45	41	61	53	52	74	60	54	36	55	42	41	50	71	35	38	79
TCONS_00009975.p1_S1603	hypothetical protein AWC38_SpisGene3943 [Stylophora pistillata]	100	100	100	100	0	100	100	92	100	100	0	0	100	100	100	100	100	100	100	100	100	100	0	100	100	100	10	100	100	100	100	100	100
TCONS_00010302.p1_S1606	-	100	100	100	69	100	100	100	100	1	100	100	0	100	100	100	100	40	1	100	100	100	100	100	100	100	0	0	0	100	38	100	2	100
TCONS_00010739.p1_S1606	uncharacterized protein LOC111339393 [Stylophora pistillata]	13	100	100	100	0	0	100	100	100	100	100	15	100	100	100	100	100	100	100	21	100	100	100	100	3	100	100	100	100	100	100	100	100
TCONS_00010841.p1_S1601	putative periplasmic serine endoprotease DegP-like [Stylophora pistillata]	100	100	100	100	100	100	100	93	93	0	88	76	100	100	100	100	100	100	100	100	100	100	100	100	100	100	100	100	100	100	100	100	100
TCONS_00010963.p1_S1601	hypothetical protein AWC38_SpisGene7872 [Stylophora pistillata]	0	9	100	100	12	0	100	2	7	0	100	4	100	1	4	100	0	100	0	1	100	100	100	100	6	0	0	0	0	6	23	0	13
TCONS_00011057.p1_S1603	hypothetical protein AWC38_SpisGene8879 [Stylophora pistillata]	100	0	0	0	100	1	0	0	100	0	0	0	9	100	0	0	100	0	0	0	0	100	100	0	0	0	0	0	4	100	100	0	0
TCONS_00011654.p1_S1601	uncharacterized protein LOC110246679 [Exaiptasia pallida]	45	100	100	100	100	100	75	100	57	18	100	37	100	100	100	100	62	100	52	100	100	100	20	100	100	100	100	25	100	100	100	62	64
TCONS_00011819.p1_S1603	uncharacterized protein LOC110058389 [Orbicella faveolata]	5	0	0	0	0	100	0	1	100	0	0	100	5	8	100	100	81	100	5	100	0	0	7	7	1	7	0	100	100	0	100	0	0

Supporting Table S5

Table S5. Genome or transcriptome data used for blast databases in this study

BLAST database	Kingdom	-	Phylum	Class	Order	Family	Genus	Species	RefSeq Assembly ID/URL
Cnidaria database 1	Metazoa	Eumetazoa	Cnidaria	Hydrozoa	Anthoathecata	Aplanulata	*Hydra*	*vulgaris*	GCF_000004095.1
	Metazoa	Eumetazoa	Cnidaria	Anthozoa	Actiniaria	Actiniidae	*Nematostella*	*vectensis*	GCF_000209225.1
	Metazoa	Eumetazoa	Cnidaria	Anthozoa	Actiniaria	Actiniidae	*Anthopleura*	*elegantissima*	http://people.oregonstate.edu/~meyere/data.html
	Metazoa	Eumetazoa	Cnidaria	Anthozoa	Actiniaria	Aiptasiidae	*Exaiptasia*	*pallida*	GCF_001417965.1
	Metazoa	Eumetazoa	Cnidaria	Anthozoa	Scleractinia	Astrocoeniina	*Madracis*	*auretenra*	http://people.oregonstate.edu/~meyere/data.html
	Metazoa	Eumetazoa	Cnidaria	Anthozoa	Scleractinia	Astrocoeniina	*Seriatopora*	*hystrix*	http://people.oregonstate.edu/~meyere/data.html
	Metazoa	Eumetazoa	Cnidaria	Anthozoa	Scleractinia	Faviina	*Montastraea*	*cavernosa*	http://people.oregonstate.edu/~meyere/data.html
	Metazoa	Eumetazoa	Cnidaria	Anthozoa	Scleractinia	Faviina	*Orbicella*	*faveolata*	GCF_002042975.1
	Metazoa	Eumetazoa	Cnidaria	Anthozoa	Scleractinia	Faviina	*Pseudodiploria*	*strigosa*	http://people.oregonstate.edu/~meyere/data.html
	Metazoa	Eumetazoa	Cnidaria	Anthozoa	Scleractinia	Faviina	*Platygyra*	*daedalea*	http://people.oregonstate.edu/~meyere/data.html
	Metazoa	Eumetazoa	Cnidaria	Anthozoa	Scleractinia	Fungiina	*Fungia*	*scutaria*	http://people.oregonstate.edu/~meyere/data.html
	Metazoa	Eumetazoa	Cnidaria	Anthozoa	Scleractinia	Fungiina	*Siderastrea*	*siderea*	http://sites.bu.edu/davieslab/data-code/
Cnidaria database 2	Metazoa	Eumetazoa	Cnidaria	Anthozoa	Scleractinia	Acroporidae	*Montipora*	*aequituberculata*	https://matzlab.weebly.com/data--code.html
	Metazoa	Eumetazoa	Cnidaria	Anthozoa	Scleractinia	Acroporidae	*Montipora*	*digitata*	https://github.com/PalMuc/
	Metazoa	Eumetazoa	Cnidaria	Anthozoa	Scleractinia	Acroporidae	*Acropora*	*tenuis*	https://matzlab.weebly.com/data--code.html
Bilateria dataase	Metazoa	Eumetazoa	Arthropoda	Insecta	Diptera	Drosophilidae	*Drosophila*	*melanogaster*	GCF_000001215.4
	Metazoa	Eumetazoa	Chordata	-	-	Branchiostomidae	*Branchiostoma*	*floridae*	GCF_000003815.1
	Metazoa	Eumetazoa	Chordata	Ascidiacea	Enterogona	Cionidae	*Ciona*	*intestinalis*	GCF_000224145.2
	Metazoa	Eumetazoa	Chordata	Mammlia	Rodentia	Muridae	*Mus*	*musculus*	GCF_000001635.25
	Metazoa	Eumetazoa	Nematoda	Chromadorea	Rhabditida	Rhabditidae	*Caenorhabditis*	*elegans*	GCF_000002985.6
Sponge	Metazoa	-	Prorifera	Demospongiae	Haplosclerida	Nophatidae	*Amphimedon*	*queenslandica*	GCF_000090795.1

Table S6

Table S6. Information for high-throughput sequencing data

Sample name	SRA number	CDS average coverage
Kerama_Geruma_KrA10	DRR099374	9.8
Kerama_Geruma_KrA12	DRR099376	10.4
Kerama_Geruma_KrA13	DRR099377	9.6
Kerama_Geruma_KrA4	DRR099383	10.4
Kerama_Geruma_KrA6	DRR099385	10.3
Kerama_Yakabi_KrC10	DRR099359	9.5
Kerama_Yakabi_KrC11	DRR099360	11.7
Kerama_Yakabi_KrC12	DRR099361	10
Kerama_Yakabi_KrC13	DRR099362	11.9
Kerama_Yakabi_KrC14	DRR099363	10.1
Kerama_Yakabi_KrC15	DRR099364	9.5
Kerama_Yakabi_KrC5	DRR099369	9.8
Kerama_Yakabi_KrC7	DRR099371	10
Kerama_Yakabi_KrC8	DRR099372	12.4
Kerama_Yakabi_KrC9	DRR099373	10
Kerama_Zamami_KrE1	DRR099344	10.8
Kerama_Zamami_KrE2	DRR099345	10.8
Kerama_Zamami_KrE4	DRR099347	10
Kerama_Zamami_KrE5	DRR099348	9.6
Kerama_Zamami_KrE6	DRR099349	20.1
Kerama_Zamami_KrE8	DRR099351	15.2
Okinawa_Manza_Mz1	DRR099303	10.4
Okinawa_Ohdo_Od10	DRR099315	10.3
Okinawa_Ohdo_Od11	DRR099316	10.1
Okinawa_Ohdo_Od12	DRR099317	10.5
Okinawa_Ohdo_Od13	DRR099318	10
Okinawa_Ohdo_Od19	DRR099322	10
Okinawa_Ohdo_Od23	DRR099324	9.7
Okinawa_Ohdo_ohdo1	DRR099334	9.6
Okinawa_Ohdo_ohdo3	DRR099335	10.2

Table S7

Table S7. Primer sequences

Primer name	Primer sequence (5’-3’)
29813_LF1	CAGGTGTGAACGTAATCTCGCATGAAACGA
29813_LR1	ACTGGTKACTTTGCTTCACCTGTTGAACGC
29813_checkF1	CAAAGGCTTTCTCAGCATTTGTCGCCAAGA
29813_checkR1	ACCTGGCTTCAAATTTTGGCGCTTGCAAAC
29813_F2	TCTATAAAAACGCCGTGACAGGAAT
29813_F5	GGTTRCAAAAGGTAAAGTTCGACATG
29813_R2	CCATATTCTGGAGTATCCATTCTG
29813_R3	CGTATTCTTAGAGAGCCTTATTTCAAG
29813_checkR3	TGCAAACAATAAGTTGCTCGATCCA
29813_R4	CAGTCCGTAAAAATGCCGTGACCAA
29813_R5	TTGTGGCAAACTGCTTGACTTCGTT
29813_R6dig	TAAAAATGCCGTGACAGAGGCCTAGT
29813_R6ten	AGGGCCAATGAAACCAACAACTGAA
29813_R7ten	CTTGGGTTACTTGAGGTAATTAAGC
29813_R8ten	GAATCTCAAGTAGCCCAGGAACTT
29813_R9ten	TGATATCAAAATCGATCCGCTTGTT
51959_checkF1	CAGGGAAATAGCGCAGCARCTTGAAGATTC
51959_checkR1	GGCCTCGTTCTTTTCATMTATTTCCCTCTT
36916_LF1	CCCTGGTGTWGTTTAATTCATCTTCTAGGA
36916_LR1	AACGAGGACGAATCGCTTTCCGTTCCAGAA
36916_checkF2	AGAAGCAGTGCAGAATCACAACGTGCAGTT
36916_sF1	CAATGCTTTCTTTTCCATCGAGTT
36916_sF2	TTGTTTTCTACGAGCGATAGTCAAC
36916_sF3	TTGAACGGGGTTGATATCTGGAT
36916_sF4	GGGATRTATTTGAACCACACTCAA
36916_sF5	GCACTTTCCCATTTGATATAATTTGG
36916_sR1	TAGAAAACAAGGGCGAGTAGTCTA
36916_sR2	GCAAAATTTGGAAGAGCTAAAAGTC
36916_sR3	TTCCCTCTCAATTCGTTCTTTTTCC
34639_F1	CACACGTCCAACAGGCACTGTTCCAATA
34639_R1	CCYGGACATCGTGATTTCATCAAGAACATG
